# Impact of primary to secondary care data sharing on care quality in NHS England hospitals

**DOI:** 10.1038/s41746-023-00891-y

**Published:** 2023-08-14

**Authors:** Joe Zhang, Hutan Ashrafian, Brendan Delaney, Ara Darzi

**Affiliations:** 1 Institute of Global Health Innovation, Imperial College London, London, UK; 2 Department of Critical Care Medicine, Guy’s and St Thomas’ Hospital, London, UK

**Keywords:** Health policy, Outcomes research

## Abstract

Health information exchange (HIE) is seen as a key component of effective care but remains poorly evidenced at a health system level. In the UK National Health Service (NHS), the ability to share primary care data with secondary care clinicians is a focus of continued digital investment. In this study, we report the evolution of interoperable technology across a period of rapid digital transformation in NHS England from 2015 to 2019, and test association of primary to secondary care data-sharing capabilities with clinical care quality indicators across all acute secondary care providers (*n* = 135 NHS Trusts). In multivariable analyses, data-sharing capabilities are associated with reduction in patients breaching an Accident & Emergency (A&E) 4-h decision time threshold, and better patient-reported experience of acute hospital care quality. Using synthetic control analyses, we estimate mean 2.271% (STD+/−3.371) absolute reduction in A&E 4-h decision time breach, 12 months following introduction of data-sharing capabilities. Our findings support current digital transformation programmes for developing regional HIE networks but highlight the need to focus on implementation factors in addition to technological procurement.

## Introduction

The sharing of patient data across different healthcare locations is vital for supporting transition and continuity of care. Where primary and secondary providers have implemented electronic health records (EHR), the majority of patient medical history, treatment information, and test results, are recorded in structured data formats within software. Given the right technological conditions for interoperability, electronic flow of data between healthcare providers can help clinicians make faster and safer decisions at different time-points and locations of a patient’s journey^1^. The potential benefits are well described. For example, electronic summarisation of secondary care admissions may enable continuity of care in the community^2^, while the availability of test results can avoid unnecessary repeat investigations^3^. For patients who receive care across multiple geographically and administratively distinct providers, electronic data-sharing ensures that treatment can be properly coordinated, and that individual decision-making processes can be fully informed^4^. In particular, the provision of primary care medical history to emergency clinicians can support timely treatment decisions^5^, while a lack of suitable information transfer can be a source of deficits in emergency care coordination^6,7^.

These plausible benefits have led to healthcare provider interoperability becoming a strategic priority area for digital transformation across the world^8–10^. Investigations of clinical impact from health information exchange (HIE) have found benefit amongst specific use-cases, including to clinician and patient experience^11^, cost-effectiveness^12–14^, emergency care quality^15,16^, patient safety^17^, and investigation reduction^18,19^. However, a focus on single providers or small provider networks, and heterogeneity amongst individual technological implementations^20–22^, make comparison and generalisation across a whole healthcare system more difficult. Evidence from system-level research is more limited. Studies of the 2007 American Health Association survey found HIE to be associated with higher patient hospital ratings^23^, with HIE adoption within the study population at 10%^23^.

Within the UK National Health Service (NHS), digital transformation relies on technology procurement by local providers or regional bodies, but is driven by central policy and incentives^24^. As a result, while new software and interoperability-enabling technologies are implemented in a patchwork landscape of different system vendors, the resulting data-sharing networks are built on common functions. These include the ability to share data items from primary care EHR to local secondary care clinicians. This capability is of considerable importance because primary care providers act as a central coordinator of long-term care in the NHS, with primary care EHR hosting patients’ entire summary medical history. Through this common digital transformation model, we aim to study data-sharing impacts across a national health system.

In this study, we characterise the landscape and progression of data-sharing networks in NHS England from 2015 to 2022, and test the impact of primary to secondary care data-sharing capabilities on clinical quality indicators related to emergency care, patient experience, patient safety, and mortality. We perform our analysis across 135 Acute NHS Trusts that provide emergency and general secondary care services to local geographic areas. In the context of a growing focus on shared care records infrastructure, we provide recommendations on this aspect of digital policy.

## Results

### Evolution of local data-sharing networks in NHS England

**Fig. 1:**
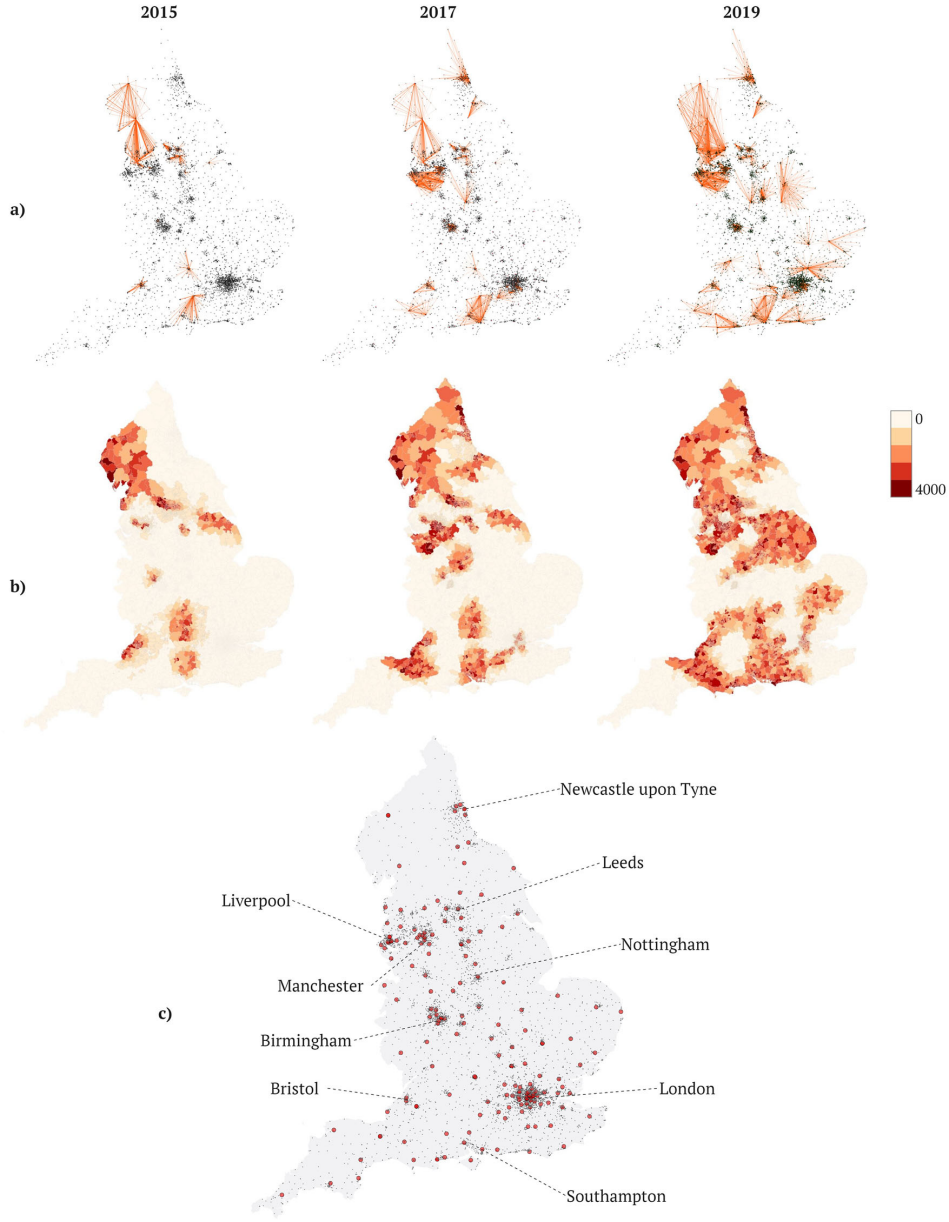
Progression of data-sharing networks and population coverage in NHS England for years 2015, 2017, and 2019. **a** Shows data-sharing relationships (orange lines) from primary to secondary care providers. **b** Shows population acute attendances at Acute Trusts with data-sharing capabilities for years 2015, 2017 and 2019, at the level of middle-layer small output areas. **c** Shows major metropolitan regions in England, marking locations of Acute NHS Trusts (red dots).

We identified three stages of infrastructure change in England. Prior to 2019 (Fig. 1a), data-sharing networks developed between individual secondary care Trusts and their local primary care providers, through adoption of one of two technological models: provision of secure remote access to local primary care views via an EHR portal (*n* = 40 at start of 2019), or centralisation of local primary care data into a hospital controlled HIE data warehouse (*n* = 22). This phase supported growth in hospital catchment of patients with accessible primary care data from 5,942,682 patients at the start of 2015 (11.3%) to 15,807,805 (29.7%) in 2017, to 27,090,091 in 2019 (49.5%) (Fig. 1b).

By the end of 2020, the majority of solutions were procured at the level of commissioning groups which administer and serve as payers for all hospital Trusts and primary care providers in a region. This resulted in centralisation into unified Local Care Records supported through a primary technology vendor (Supplementary Fig. 1), with increasing availability of hospital record sharing through common data standards. In 2023, following the allocation of funding to larger geographic regions containing populations between 2 and 10 million patients (Supplementary Fig. 1), there is expected unification of most Local Care Records into consolidated Local Health and Care Record Exemplars. This is expected to result in complete population coverage over England. Specific technological models are described in Supplementary Fig. 2.

Across different vendors and technologies, we found primary care to secondary care data-sharing to be a consistent capability, presenting standardised data across primary care providers. This common capability is therefore evaluated in our analysis.

### Population characteristics

**Fig. 2:**
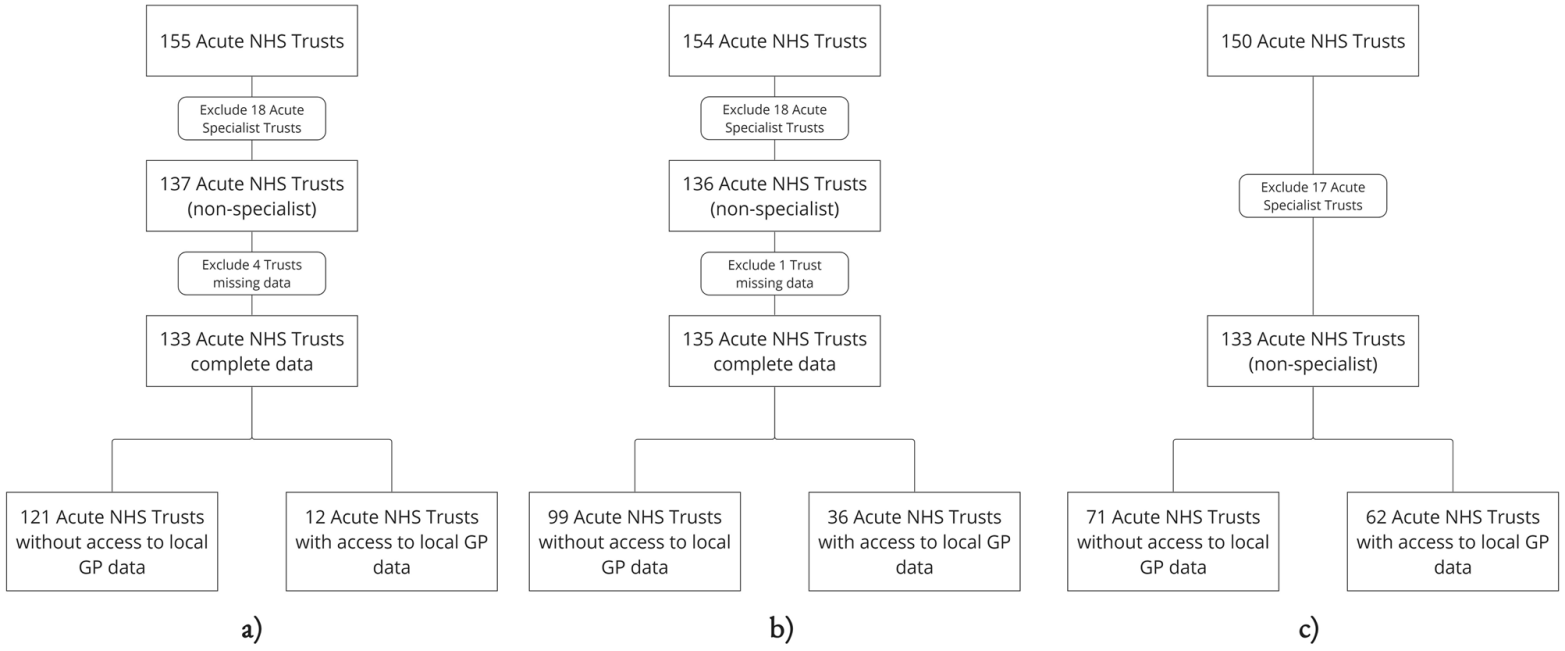
Inclusion flow diagram for Acute NHS Trusts in multivariable regression analyses of data-sharing capabilities. **a**–**c** Show analyses for the years 2015, 2017, and 2019 inclusive. For each year, Trusts are included if they have data-sharing technology implemented at the start of the year.

Because of mergers, the Acute NHS Trust population eligible for analysis ranged from 133 to 135 Trusts across time periods. Inclusion flow-charts are shown in Fig. 2. Population characteristics across analysed years are presented in Supplementary Table 1, and covariates described in Supplementary Table 2.

#### Impact of data-sharing capabilities on A&E breach percentage

Accident & Emergency (A&E) department breach is a standardised indicator describing the percentage of patients attending A&E who were not admitted, transferred, or discharged within a national 4-h target time. When adjusted for characteristics of each Trust and the attending patient population for that year, ability to share data from primary to secondary care was associated with lower A&E breach percentage in three tested years (2015: coef −3.080, 95% CI −5.646 to −0.515, *p* = 0.019; 2017: coef −3.214, 95% CI −5.904 to −0.524, *p* = 0.020; 2019: coef −2.890, 95% CI −6.138 to 0.358, *p* = 0.081). *R*esults of univariate and multivariable linear regression analyses are presented in Table 1.

**Table 1:** Results of univariate and multivariable analyses showing association of data-sharing capabilities and other organisation characteristics with Emergency Department (ED) four-hourly breach percentage in 2015, 2017, and 2019, excluding co-variates with substantial multicollinearity (as measured by Variance Inflation Factors (VIF)).

	UNIVARIATE				MULTIVARIABLE				
2015	coeff	*p* value	ci_lower	ci_upper	coeff	*p* value	ci_lower	ci_upper	VIF
const					3.504	0.605	−9.890	16.899	
Data-sharing capability	−3.442	0.021	−6.361	−0.523	−3.080	0.019	−5.646	−0.515	1.10
CDMI 2015/2016	−0.014	0.227	−0.037	0.009	−0.020	0.058	−0.041	0.001	1.26
Type 1 A&E attendance	0.106	0.338	−0.112	0.324	−0.839	0.000	−1.183	−0.494	3.69
Bed occupancy (%)	0.178	0.027	0.021	0.335	0.099	0.168	−0.042	0.241	1.17
Emergency activity	0.090	0.000	0.044	0.136	0.203	0.000	0.099	0.306	6.75
Elective activity	0.038	0.017	0.007	0.068	−0.034	0.305	−0.099	0.0310	6.49
General & acute referrals	0.034	0.001	0.014	0.054	0.040	0.046	0.001	0.080	5.16
Number of nurses	0.131	0.018	0.023	0.240	−0.068	0.598	−0.322	0.186	7.84
Number of managers	0.012	0.068	−0.001	0.025	0.009	0.280	−0.007	0.025	2.16
Population deprivation	0.000	0.996	−0.044	0.044	0.026	0.214	−0.015	0.067	1.29
Foundation Trust status	−3.384	0.000	−4.988	−1.780	−2.480	0.002	−4.000	−0.960	1.18
Academic Trust status	0.406	0.709	−1.745	2.557	0.335	0.793	−2.191	2.861	2.05
2017	coeff	pval	ci_lower	ci_upper	coeff	pval	ci_lower	ci_upper	VIF
const					−9.269	0.459	−33.992	15.455	
Data-sharing capability	−3.529	0.017	−6.407	−0.652	−3.214	0.020	−5.904	−0.524	1.18
Global Digital Exemplar	−1.277	0.541	−5.397	2.843	−1.163	0.596	−5.494	3.169	1.56
CDMI 2017	−0.003	0.891	−0.039	0.034	−0.022	0.233	−0.057	0.014	1.36
Wannacry impacted	2.644	0.122	−0.720	6.009	2.648	0.079	−0.315	5.610	1.08
Type 1 A&E attendance	0.266	0.097	−0.049	0.581	−1.059	0.001	−1.648	−0.469	4.85
Bed occupancy (%)	0.403	0.003	0.144	0.661	0.275	0.030	0.028	0.523	1.21
Emergency activity	0.130	0.000	0.065	0.196	0.216	0.004	0.072	0.359	6.05
Elective activity	0.072	0.002	0.027	0.116	−0.017	0.746	−0.119	0.086	6.82
General & acute referrals	0.042	0.006	0.013	0.072	0.037	0.251	−0.027	0.100	6.06
Number of nurses	0.249	0.003	0.087	0.412	0.042	0.842	−0.378	0.463	8.85
Number of managers	0.020	0.061	−0.001	0.041	0.004	0.795	−0.024	0.032	2.40
Population deprivation	0.067	0.055	−0.001	0.135	0.110	0.002	0.043	0.177	1.35
Foundation Trust status	−4.510	0.001	−7.017	−2.003	−3.348	0.006	−5.717	−0.979	1.15
Academic Trust status	1.775	0.301	−1.607	5.157	0.146	0.947	−4.170	4.462	2.29
2019	coeff	pval	ci_lower	ci_upper	coeff	pval	ci_lower	ci_upper	VIF
const					−10.817	0.553	−46.792	25.157	
Data-sharing capability	−3.474	0.043	−6.830	−0.118	−2.890	0.081	−6.138	0.358	1.17
Global Digital Exemplar	−4.567	0.107	−10.139	1.004	−8.479	0.006	−14.464	−2.495	1.46
Type 1 A&E attendance	0.134	0.462	−0.226	0.495	−1.359	0.000	−2.082	−0.635	5.16
Bed occupancy (%)	0.537	0.006	0.158	0.916	0.311	0.108	−0.069	0.692	1.22
Emergency activity	0.085	0.013	0.018	0.152	0.235	0.004	0.078	0.393	6.77
Elective activity	0.053	0.043	0.002	0.103	−0.013	0.832	−0.137	0.111	7.42
General & acute referrals	0.040	0.040	0.002	0.077	0.070	0.098	−0.013	0.154	6.09
Number of nurses	0.139	0.123	−0.038	0.316	−0.048	0.846	−0.539	0.442	9.70
Number of managers	−0.004	0.720	−0.027	0.019	−0.022	0.166	−0.053	0.009	2.42
Population deprivation	0.077	0.103	−0.016	0.170	0.121	0.011	0.028	0.214	1.26
Foundation Trust	−2.302	0.180	−5.681	1.076	−0.755	0.642	−3.968	2.457	1.15
Academic Trust status	2.307	0.287	−1.963	6.578	4.698	0.116	−1.180	10.575	2.42

Co-variates are described in Supplementary Table 2.

Results of univariate and multivariable analyses showing adjusted association of data-sharing capabilities with Emergency Department (ED) 4-hourly breach percentage.

*CDMI* Clinical Digital Maturity Index, *A&E* Accident and Emergency.

**Fig. 3:**
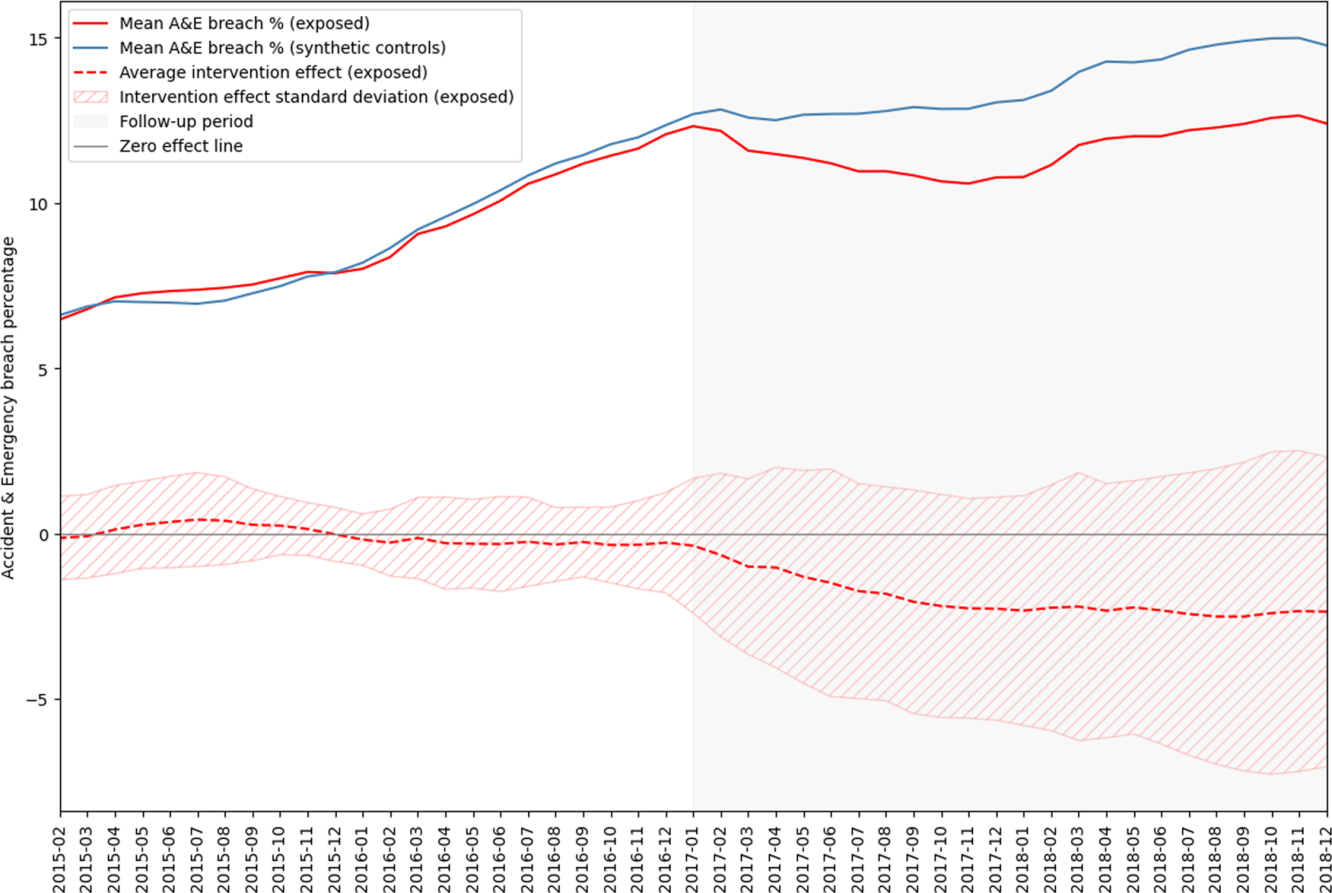
Average breach percentage and intervention effect in case vs control Trusts over time. A&E breach percentage over time in Trusts acquiring data-sharing capabilities (*n* = 15, red solid line) vs synthetic controls (blue solid line) constructed from a group of Trusts with no data-sharing capabilities throughout the experimental period (*n* = 71). Average treatment effect in intervention Trusts (with standard deviation) shown with dotted line. Matching includes time-varying covariables at monthly resolution: (1) emergency department attendance; (2) bed occupancy; (3) case-mix; (4) deprivation characteristics of attending population; (5) nurses per bed. Matching performed within groups of Trusts with same NHS classification of size and function, prior to pooling results.

To explore effect size and trajectory over time, we analyzed the impact of exposure to a new data-sharing intervention during the year 2016 (exposed *n* = 15) (Supplementary Fig. 3). When matched on historic time-varying characteristics to synthetically generated controls, exposed Trusts demonstrated relative lower breach percentage that was sustained over time (Fig. 3), with average “treatment” effect (ATE) in the exposed population of −1.492 at 6 months (STD+/−3.443, vs placebo ATE of −0.706, STD+/−4.395, *p* = 0.227), −2.271 at 12 months (STD+/−3.371, vs placebo ATE of −0.840, STD+/−4.846, *p* = 0.091), and –2.322 at 18 months (STD+/−4.047, vs placebo ATE of –0.551, STD+/−5.195, *p* = 0.079). Placebos are generated for comparison of treatment effect using the control population (see Methods). Per-month outcomes and matching fit shown in Supplementary Table 3 and Supplementary Fig. 4.

Technological acquisition does not guarantee implementation success. Acute Trust responses to a national digital maturity assessment in 2017 (the Clinical Digital Maturity Index or CDMI) showed varying self-assessment of data-sharing function, despite acquiring such technology during 2016. In sensitivity analysis to consider possible impacts from non-technological implementation factors, we restricted cases to those that reported improved functional interoperability in the 2017 survey (*n* = 11, 73% of original cases). ATE in these Trusts, when matched to synthetic controls, was −1.493 at 6 months (STD+/−2.429, vs placebo ATE of −0.067, STD+/−3.296, *p* = 0.053), −2.227 at 12 months (STD+/−3.827, vs placebo ATE of –0.242, STD+/−4.125, *p* = 0.068), and –3.059 at 18 months (STD+/−4.517, vs placebo ATE of –0.149, STD+/−4.761, *p* = 0.035). Results are presented in Supplementary Fig. 5 and Supplementary Table 4.

### Impact on patient experiences of emergency care

The NHS national patient survey collects response data across multiple care quality domains. Data-sharing capabilities demonstrated significant adjusted association with better patient experiences of urgent and emergency care across biennial survey iterations (Survey A 16/17: coef 1.579, 95% CI 0.290 to 2.868, *p* = 0.017; Survey B 18/19: coef 1.319, 95% CI 0.075 to 2.562, *p* = 0.038) (Table 2).

**Table 2:** Results of univariate and multivariable analyses showing association of data-sharing capabilities and other organisation characteristics with measured patient experience of emergency care quality across two surveys (A: 2016/2017, and B: 2018/2019), excluding co-variates with substantial multicollinearity (as measured by Variance Inflation Factors (VIF)).

	UNIVARIATE				MULTIVARIABLE				
Survey A	coeff	*p* value	ci_lower	ci_upper	coeff	*p* value	ci_lower	ci_upper	VIF
const					79.344	0.000	66.821	91.867	
Data-sharing capability	1.420	0.038	0.077	2.764	1.579	0.017	0.290	2.868	1.17
Global Digital Exemplar	0.698	0.470	−1.208	2.605	0.887	0.404	−1.214	2.989	1.59
CDMI 2017	0.006	0.478	−0.011	0.023	0.007	0.454	−0.011	0.024	1.39
Wannacry impacted	0.412	0.605	−1.161	1.984	0.237	0.744	−1.194	1.667	1.08
Type 1 A&E attendance	−0.320	0.000	−0.460	−0.181	−0.151	0.268	−0.419	0.118	4.15
Bed occupancy (%)	−0.083	0.228	−0.218	0.052	−0.065	0.318	−0.194	0.064	1.18
Emergency activity	−0.053	0.002	−0.086	−0.020	0.009	0.821	−0.067	0.085	6.48
Elective activity	−0.023	0.043	−0.045	−0.001	0.021	0.413	−0.030	0.072	6.89
General & acute referrals	−0.026	0.000	−0.040	−0.013	−0.031	0.032	−0.060	−0.003	5.20
Number of nurses	−0.100	0.014	−0.180	−0.021	0.090	0.404	−0.122	0.302	8.88
Number of managers	−0.007	0.197	−0.017	0.004	−0.002	0.795	−0.016	0.012	2.39
Population deprivation	−0.053	0.001	−0.084	−0.023	−0.054	0.001	−0.087	−0.022	1.36
Foundation Trust status	1.574	0.010	0.377	2.772	1.022	0.085	−0.144	2.188	1.18
Academic Trust status	−1.249	0.115	−2.808	0.309	−1.797	0.094	−3.908	0.314	2.36
Survey B	coeff	pval	ci_lower	ci_upper	coeff	pval	ci_lower	ci_upper	VIF
const					94.382	0.000	79.622	109.143	
Data-sharing capability	1.356	0.043	0.041	2.670	1.319	0.038	0.075	2.562	1.13
Global Digital Exemplar	0.934	0.382	−1.173	3.041	0.610	0.598	−1.678	2.898	1.53
Type 1 A&E attendance	−0.270	0.001	−0.425	−0.114	−0.083	0.581	−0.381	0.214	4.35
Bed occupancy (%)	−0.238	0.003	−0.394	−0.083	−0.205	0.011	−0.361	−0.048	1.22
Emergency activity	−0.044	0.006	−0.074	−0.013	−0.030	0.356	−0.095	0.034	5.44
Elective activity	−0.015	0.182	−0.038	0.007	0.014	0.602	−0.038	0.066	6.71
General & acute referrals	−0.020	0.009	−0.035	−0.005	−0.029	0.068	−0.061	0.002	5.54
Number of nurses	−0.056	0.157	−0.133	0.022	0.176	0.098	−0.033	0.385	9.34
Number of managers	−0.00	0.956	−0.010	0.009	−0.004	0.603	−0.017	0.010	2.58
Population deprivation	−0.063	0.001	−0.098	−0.027	−0.070	0.000	−0.105	−0.034	1.18
Foundation Trust status	1.422	0.034	0.113	2.731	0.536	0.397	−0.713	1.785	1.14
Academic Trust status	−0.625	0.465	−2.314	1.063	−1.021	0.382	−3.324	1.283	2.41

Co-variates are described in Supplementary Table 2.

Results of univariate and multivariable analyses showing adjusted association of data-sharing capabilities with measured patient experience of emergency care quality.

*CDMI* Clinical Digital Maturity Index, *A&E* Accident and Emergency.

We again tested assumptions for implementation success in a sensitivity analysis by modifying the case group using 2017 CDMI responses. In Trusts reporting positive interoperability functionality (*n* = 32, 89% of original cases), we found association with positive patient experience from the corresponding survey iteration (Survey A 16/17: coef 1.694, 95% CI 0.335–3.053, *p* = 0.015). Results presented in Supplementary Table 5.

#### Impact on patient safety incidents and summary mortality index

We discovered no association between primary to secondary care data-sharing capabilities and either a standardised hospital mortality index, or incidence of patient safety events (adjusted per 1000 bed days), in any of the three analyzed time periods. Results of univariate and multivariable analyses are presented in Supplementary Tables 6 and 7.

Across other covariates, we found high bed occupancy to be associated with increased mortality index across multiple years, while better staffing and academic centre status were associated with reduced mortality index. In the year affected by the *Wannacry* ransomware attack, impact from the attack was associated with increased mortality index (2017: coef 7.282, 95% CI 3.429 to 11.135, *p* < 0.001). We found significant associations between population deprivation and increased patient safety event incidence in two of the analyzed years (2015: coef 0.225, 95% CI 0.041–0.409, *p* = 0.017; 2017: coef 0.265, 95% CI 0.025 to 0.505, *p* = 0.031).

## Discussion

We have described the progression of national data-sharing networks in NHS England and evaluated clinical impacts at multiple stages of this evolution. Significant effects were discovered in emergency care pathways, where primary to secondary care data-sharing capabilities were associated with reduced A&E breach percentage and improved patient experience.

Effects on these pathways are plausible, when considering previous studies of data-sharing in emergency care^25^, and the practical need for historical patient data during emergency decision-making. A&E breach percentage serves as a nationally important outcome indicator for pathway efficiency that associates with adverse outcomes including mortality^26^, and previously used to financially penalise Trusts based on performance^27^. The 2.27% absolute reduction in breach percentage at 12 months, as seen in our synthetic control experiment (Fig. 3), is equivalent to approximately 330,000 breached attendances to NHS England Acute Trust A&Es in a given year (taking 15 million attendances in 2018/2019 prior to the COVID-19 pandemic) although significant caveats certainly apply considering the wide uncertainty interval of the estimate. Impact on improved patient experience of emergency care is similarly plausible through better informed clinicians, reduction in unnecessary investigation replication^19^, and the likelihood of patient experience being impacted by the same pathway inefficiencies that are measured by indicators such as A&E breach.

No impacts were found in analysis of patient mortality or safety incidents as outcomes. Existing evidence suggests association between interoperability and a reduction in patient safety events^17^. Our lack of positive findings could be explained by differences in the outcome variable. For example, previous studies have discovered specific interoperability-related events from patient safety reports^28,29^, and it is likely that these impacts are lost in aggregate national safety data. Previous studies that examined impacts on direct harm and mortality from HIE^30,31^ and organisational digital maturity^32^ also did not discover significant associations. It is possible that such mortality indicators, while worth exploring, are too multi-factorial to be considered useful measurements of outcome from broad digital interventions. The association of *Wannacry* ransomware with a hospital mortality index bears further investigation, particularly considering a previous study discovering no effect on mortality (but investigating only the initial infection week and mortality statistics from A&E^33^).

Compared to previous analyses of national interoperability in the United States^23,26^ and digital maturity in the NHS^32^, our study presents a number of strengths. First—rather than a binary measure of participation that represents potentially heterogeneous HIEs, we look at directional provision of a particular data that is common across all examined units. This also allows consideration of plausible functions from directional movement of data, and can be applied to other hypotheses—such as data movement from secondary to primary care for informing primary care mediated indicators such as patient follow-up and preventable readmission. Second—rather than a cross-sectional study at one point in time, we analyze across multiple years and in time-series, across periods of digital transformation. Third—our study explores a wider range of clinical and patient-centred outcomes and adjusts for dynamic organisation, load, and population covariables (rather than fixed organisation characteristics only).

We acknowledge a number of analysis limitations. Despite achieving good matching fit across synthetic control analyses, neither this method nor our multivariable analyses can account for unmeasured effects or ‘shocks’ that might affect the outcome of individual Trusts. In addition, we make no claims of causation from this analysis. There may be unmeasured confounding that result from both complexity of digital intervention implementation, and other factors that may impact on clinical indicators. While the majority of data in this study uses standardised collection and derivation methodology, the use of patient safety events as an outcome also depends on uniformity in safety reporting across Trusts. Additionally, while we used statutory reporting requirements to map data-sharing capabilities, it is possible that some interventions could not be discovered due to incorrect Trust responses. Finally, we note two effects that may be insufficiently considered. First is the ‘implementation layer’ that includes workforce training, workflow integration, and usability, which may serve to increase functional efficacy of data-sharing technology^34,35^. We considered this using Trust self-assessment data, but a qualitative approach would be required to sufficiently measure this. Second is an unmeasured network effect as regional interoperability improves outside of the analysis population to community, mental health, and ambulance organisations. This would plausibly increase the quantity and quality of data accessible to Trusts with data-sharing technologies. Both of these considerations may be reflected in increasing effect size seen over time. These limitations reflect common difficulties in evaluating complex healthcare interventions, particularly in digital health. Our approach is aligned to the UK Medical Research Council framework for complex systems research, when approaching from the perspective of effectiveness at a programme level^36^. However, as discussed by Skivington et al, translation of research evaluation to impactful implementation requires additional work within specific contexts, which includes understanding the capabilities and requirements of local stakeholders and organisations, and exploring economic features of the local system.

While such local analyses are outside the scope of this work, we can contextualise our findings within on-going digital transformation in the NHS. Despite earlier maturity in primary to secondary care data-sharing, heterogeneity across HIE and EHR vendors has historically made hospital data interoperability a greater challenge^4,37^. In 2023 and beyond, data-sharing will instead be structured around NHS England regions with populations of millions, with a main technology supplier incorporating multiple local data flows through use of common data standards. New regional data-sharing agreements are opening the door to secondary uses, with a move towards permanent population data warehousing in some regions. In any healthcare system that advances to these mature levels of interoperability, there are a number of implications:

1. Beyond demonstrating potential benefits from data provision, we must now focus on optimising the translational layer between data-sharing capabilities and clinical workflow improvements. A focus on systems usability and workflow positioning is key to helping technologically heterogeneous providers achieve uniform impact. This requires close involvement of end-users in co-designing interfaces and processes that might directly enhance quality and safety within their clinical pathways.
2. While this study adds evidence for specific impacts of data-sharing interventions, overall benefits may be marginal when competing amongst other, more prosaic, determinants of pathway efficiency. We found other covariates in analysis with significance across multiple outcomes, including measures of load, staffing, and bed occupancy. In addition, deprivation of the attending population was associated with worse indicators across numerous analyses, thus supporting well-established findings in previous studies^38^. Overall, investment into interoperability will see the most gains when building upon basic resourcing and staffing requirements.
3. Increasingly, data for clinical use and data for secondary use rely on the same EHR extractions and the same data flows. This is a unique opportunity for interoperable data to be enriched by automated analysis and decision support, generating greater value from established data-sharing infrastructure^39^. This would be a foundation for a Learning Health System where population data flows can lead to continual process evolution. Direct interfacing with clinicians can incentivize higher quality data entry^40^.
4. Proliferation of data flow across LHCREs (on top of existing data flows for secondary uses such as reimbursements and population research) will unavoidably increase privacy risk. Robust information governance procedures, designed in consultation with patients and public, are essential. At present, the NHS operates on an ‘opt-out’ basis at the point of extraction for different data uses^41^. Evolving uses of data, that include numerous secondary use-cases beyond the point of extraction, mean that any ‘opt-out’ process is better placed at the level of usage, rather than the level of extraction.

In conclusion, implementation of primary to secondary care data-sharing in NHS England is associated with positive impacts within emergency care pathways. Developments over the past decade in data-sharing infrastructure have great potential for transforming care quality. Future work must focus on the best practices that enable translation of data-sharing technology to clinical impact, as well as effective and safe secondary uses for patient data.

## Methods

### Study design and setting

A retrospective longitudinal analysis was conducted using data from NHS England Acute Hospital Trusts. Our primary aim was to assess the association of data-sharing capabilities between a Trust and its local primary care providers, with care quality indicators. As a secondary aim, and to provide the independent variables for this analysis, we characterise the progression of data-sharing capabilities in England between 2015 and 2022.

### Describing data-sharing capabilities

No central standard record exists of technology or data-sharing capability acquisition in the NHS (limiting previous studies of data-sharing networks in the UK^4^). We sent information disclosure requests in February 2022 (under the Freedom of Information Act 2000) to dedicated information teams at 152 Acute NHS Trusts, to identify procurement of historical and contemporaneous data-sharing technologies (see Supplementary Table 8). Under the Freedom of Information Act, Trusts are legally obligated to provide any recorded information on the subject requested^42^. In three Trusts that no longer exist due to mergers/closures (and as a cross-reference step for other Trusts) we reviewed all Trust annual reports between the years 2015 and 2022 for references to HIT procurement. We map data flows between primary and secondary care providers over the period 2015 to 2022 and identify regional system suppliers. Population inclusion in data-sharing networks over the study period is described using per-hospital catchment from small geographic areas for each year.

### Data sources

Covariate and outcome data used in this study are taken from national aggregate secondary care datasets. These are derived by central NHS organisations from administrative patient-level data collections^43^, nationally administered patient surveys^44^, and centralised patient safety reporting^45^. Data sources are described in Supplementary Table 2. Data was obtained at monthly time resolution from January 2015 to January 2019. Missing data were found to represent periods that Trusts were non-operational, or where Trusts ceased to exist due to mergers and closures. These Trusts were excluded.

For sensitivity analysis, we used results from the NHS Clinical Digital Maturity Index^46^, a health information technology survey across all NHS England Trusts that includes self-ratings of interoperability function measured across two survey iterations.

### Population and exposure

Our population included all NHS Acute Hospital Trusts (those providing acute general hospital services). We excluded Specialist Acute Trusts that do not offer acute general services, and Trusts if they were non-operational at any point during each analysis period.

In multivariable analyses, “cases” were defined as Trusts which had procured primary to secondary care data-sharing capabilities by the start of the analyzed year. In synthetic control analyses, “cases” were defined as Trusts that acquired such capabilities during the year 2016 (the intervention period), while a control group was composed of Trusts which did not possess or obtain any such capabilities during the entire study period, including a two-year follow-up period. We ended follow-up in 2019, prior to encountering effects from the COVID-19 pandemic on hospital admissions, pathways, and outcomes.

### Outcome indicators

We chose outcomes for high clinical importance, plausibility in being affected by a data-sharing exposure, and for relevance as national performance indicators. Chosen outcomes were:

1. A&E breach—a measure of patients that fall outside of NHS A&E 4-h care standards, seen as a key indicator for care quality and performance^47^;
2. Patient experience—measured using the biennial National Patient Survey^44^ of urgent and emergency care using aggregate scores across themes of access to and quality of care.
3. Summary Hospital-Level Mortality Index (SHMI)—measure of deaths in hospital or within 30 days of discharge adjusted for expected risk of death based on national population data^48^;
4. Patient safety incidents from the National Reporting and Learning System^45^ related to patient admission and clinical assessment, adjusted for bed days.

Additional outcome information is found in Supplementary Table 2. Outcomes (1), (3), and (4) are standardised performance indicators produced annually from patient-level data. For outcome (2), we analysed results from two survey iterations, termed Survey A (2016/2017) and Survey B (2018/2019). Survey A was conducted across 136 Acute NHS Trusts and received 41,941 responses (median 309, interquartile range 254 to 354). Survey B was conducted across 133 Acute NHS Trusts, receiving 42,707 responses (median 315.5, interquartile range 272 to 362).

### Trust covariates

Covariates were chosen for their plausible association with outcomes of interest, and for established use in confounding adjustment in previous analyses of aggregate hospital-level outcomes^30,32^. These are presented in Supplementary Table 2. In short, we included: Trust classifications (including academic and Foundation Trust status); number of active and occupied beds; emergency and elective attendance, admissions, and inpatient activity; number of primary to secondary care referrals; number of active nursing, doctor, and management staff; Clinical Digital Maturity Index score over the intervention period; deprivation of attending population; special Global Digital Exemplar Trust status (carrying implications for Trust funding)^49^; and whether the Trust was affected by the *Wannacry* ransomware attack which resulted in severe downtime and financial losses for involved Trusts during the study period^50^.

### Multivariable analysis

We used multivariable linear regression to test association of exposure with outcome features adjusted for organisation characteristics. For outcomes (1), (3) and (4), we conducted analyses across three years that represent substantial progression in digital transformation and sizeable change in the exposed Trust population (2015, *n* = 12 (9%); 2017, *n* = 36 (26.7%); 2019, *n* = 62 (45.9%)). For outcome (2), we analyzed for the corresponding survey years. Covariates for adjustment were removed from the model if they exhibited severe multicollinearity (using a variance inflation factor threshold of 10). We constructed univariate models, and multivariable models testing relative association of all included co-variates. We confirmed linearity, normality, and homoscedasticity of residuals visually (Supplementary Figs. 6–9). Analysis was performed using *Python 3.7* and the *statsmodels 0.13.2* package (Supplementary Table 9).

### Synthetic control analysis

For outcome (1) we conducted further analysis using the synthetic control method pioneered by ref. ^51,52^, and used for investigating interventions such as pay-for-performance at national^53^ and hospital^54^ levels in the NHS. The aim of this additional analysis was to understand trajectory of effect size over time. As with score-based matching, this method aims to match each exposed Trust to a control. However, Trust and regional variations, as well as significance of time-varying factors, mean that direct, close matches are difficult. A synthetic control approach instead matches each exposed Trust (case) with a synthetic ‘fake’ Trust (control), created from a weighted combination of unexposed Trusts, such that the synthetic control most closely matches the exposed Trust on pre-exposure time-varying covariates and outcome. Weights are computed to minimise squared prediction error on pre-intervention data and penalised to avoid extrapolation where the sum of weights is greater than 1, or where weights are negative. The post-intervention outcome over time of the synthetic control is subsequently the weighted combination of unexposed Trust outcomes and is used as the counterfactual. This takes as an assumption that the combination of unexposed Trusts is a stable, linear representation of the exposed Trust. Additional discussion of synthetic controls for large-scale health interventions is made by ref. ^55^.

A synthetic control was generated for each exposed Trust, from groups of unexposed Trusts with the same NHS classification of Trust size: smaller district hospitals (offering general services) or larger multi-site Trusts (offering additional tertiary referral services). Matching within groups aims to create greater homogeneity in size, staffing, specialty case-mix, and facilities, between exposed Trusts and unexposed Trusts in the ‘donor pool’. We engineered time-series features that represent on-going Trust activities with plausible impact on the measured outcome, also considering feature significance in multivariable analyses. These included occupancy (ratio of occupied general beds to available general beds per month), A&E load (mean emergency attendances per month), case-mix (ratio of emergency to elective cases per month), deprivation of attending population (monthly admissions from bottom two index of multiple deprivation quintiles), and nurse staffing to bed ratios. We used a 12-month rolling average for all indicators to account for seasonal variation. As cases included all Trusts exposed during 2016, we set the exposure point to the 31st of December 2016. We computed a synthetic control for each exposed Trust using pre-treatment data (2015 and 2016) and calculated subsequent intervention effect (difference between observed outcome and synthetic counterfactual) over a follow-up period of two years (2017 and 2018). Analyses were performed using *Python 3.7* and the *SyntheticControlMethods 1.1.17* package (Supplementary Table 9).

To create an estimate of intervention significance across the cohort, we used a ‘placebo’ method: generating a synthetic control for all Trusts in the control group to estimate incidental effects in these Trusts that did not receive an intervention. We compared pooled intervention effects of exposed Trusts, with pooled incidental intervention effects of control Trusts, using an independent-samples, one-tailed *t* test at each month in the follow-up period. We report mean and standard deviation of intervention effects in each pool, and p-value, at each month.

### Sensitivity analyses

Our analysis of data-sharing capabilities assumes homogeneity in implementation, including across unmeasured factors such as useful levels of uptake within each Trust. As a sensitivity analysis, we included consideration of post-implementation functionality by modifying case populations using results of the Clinical Digital Maturity Index (CDMI). Responses to this survey included a self-assessment of interoperability function, taken as a surrogate measurement for implementation maturity. As only two CDMI survey iterations exist (in 2016 and 2017), improved functionality was defined as CDMI score increment between the two surveys from a negative measure (prior to intervention) to a positive measure (after intervention).

### Ethics

As a secondary analysis of aggregate population and organisation data from government statistical datasets, this study did not require ethical approval.

### Reporting summary

Further information on research design is available in the Nature Research Reporting Summary linked to this article.

### Supplementary information

Supplementary Information

Reporting Summary

## Data Availability

Datasets analyzed in this study are available to download from data repositories owned by NHS England^56^, NHS Digital^57^, and the Office of National Statistics^58^. Patient survey outcome data available from the Care Quality Commission^59^. Patient safety incident reporting data available from the NHS Improvement^60^. Further description of data used is given in Supplementary Table 2.

## Code availability

Analyses are performed using *Python 3.7*. Statistical packages used include *pandas 1.4.4*, *numpy 1.21.5*, *statsmodels 0.13.2*, and *SyntheticControlMethods 1.1.17*. Further package details are given in Supplementary Table 9.
