# Supplementary material for: Impact of primary to secondary care data sharing on care quality in NHS England hospitals
Source: NPJ Digit Med. 2023 Aug 14;6:144. doi: 10.1038/s41746-023-00891-y (PMC10425337; doi:10.1038/s41746-023-00891-y)
Supplement: Supplementary file 1 — Supplementary Information [file 41746_2023_891_MOESM1_ESM.pdf]

Impact of primary to secondary care data sharing on care quality in NHS England hospitals

SUPPLEMENTARY INFORMATION - TABLES AND FIGURES

Joe Zhang[1,2], Hutan Ashrafian[1], Prof. Brendan Delaney[1], Prof. Ara Darzi[1]

[1]*Institute of Global Health Innovation, Imperial College London, UK*

[2]*Department of Critical Care Medicine, Guy's and St Thomas' Hospital, UK*

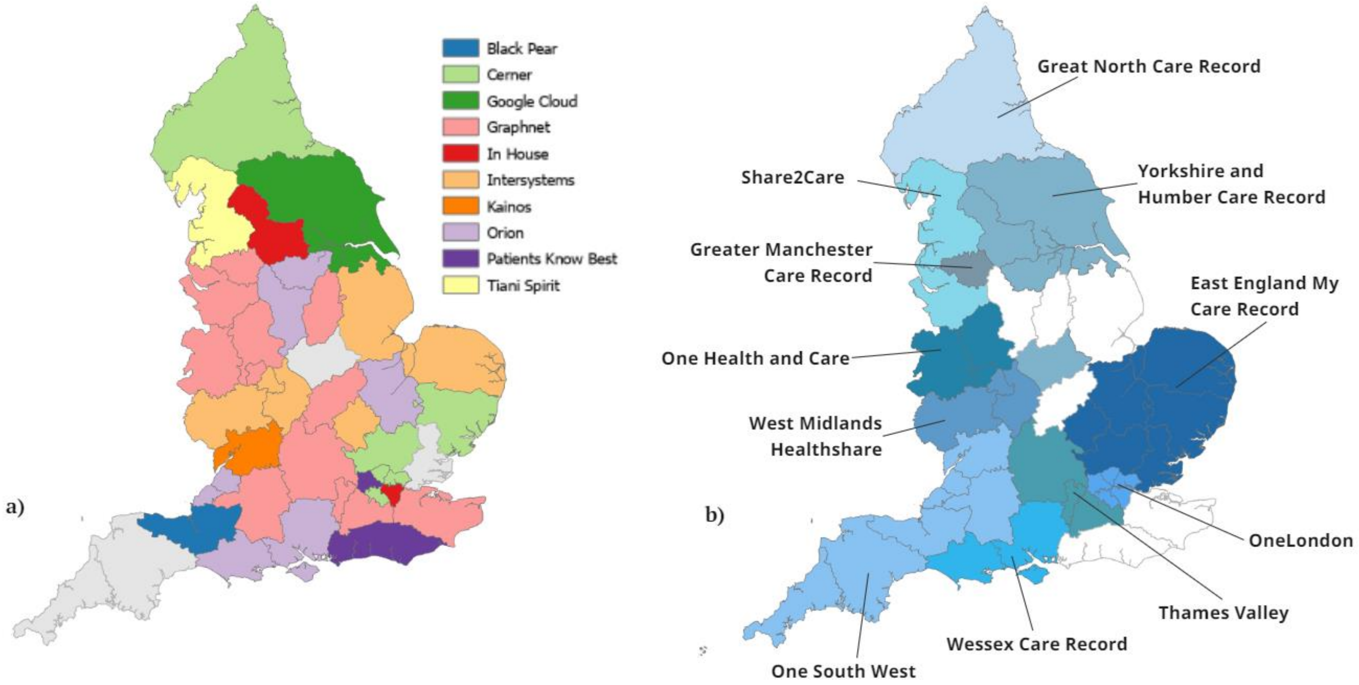

Supplementary Figure 1 – Geographic structure of commissioning and regional-level shared care record development in the NHS. Panel a) integrated care board commissioning level procurement of care records from specified vendors; Panel b) structure of Local Health and Care Records at larger geographic region level, covering populations of between 2 and 10 million patients.

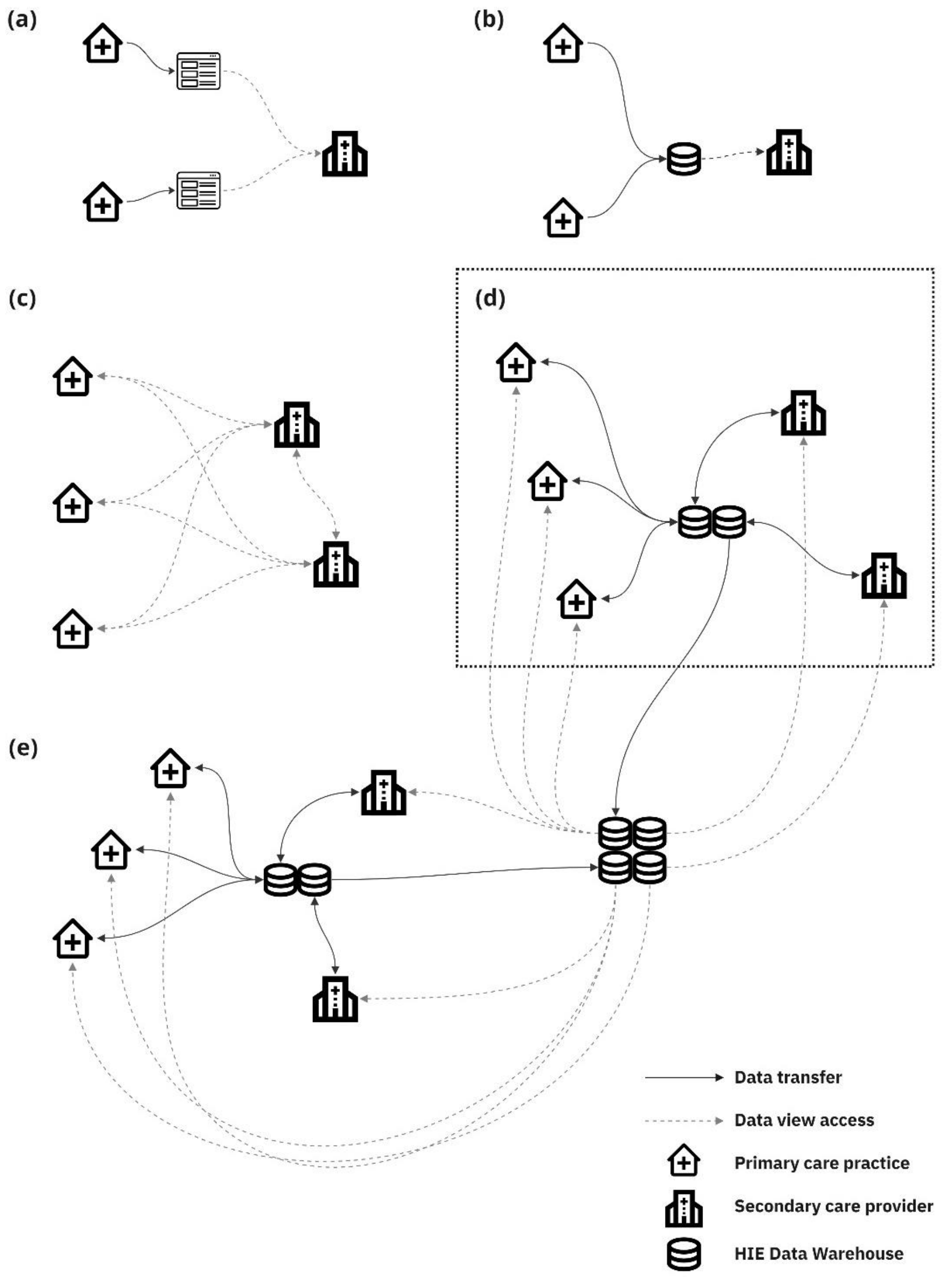

Supplementary Figure 2 – Models of primary to secondary care data-sharing in NHS England 2015 to 2022. Prior to 2019, dominant models of data-sharing included (a) remote web view of primary care records (e.g. EMIS Web Viewer) and (b) local data warehouse solution. After 2019 this was replaced by regional (c) federated or (d) centralized solutions at the level of commissioning groups. Between 2022 and 2023, multiple smaller solutions will be further centralized into (e) Health and Care Record Exemplar networks for health information exchange (HIE) across populations of 10 million patients.

	2015 (n=133)	2017 (n=135)	2019 (n=133)
SCR implementations	12 (9%)	36 (26.7%)	62 (45.9%)
Foundation Trust status	68.0 (51.1%)	82.0 (60.7%)	96.0 (72.2%)
Academic Trust status	26 (19.5%)	24 (17.8%)	23 (17.3%)
CDMI score (2015/2016 and 2017)	185.0 [163.0 - 217.0]	217.0 [188.0 - 243.0]	*NA*
Elective inpatient cases (1000s)	48.8 [33.6 - 71.0]	49.1 [33.7 - 71.2]	52.4 [35.2 - 74.4]
Emergency inpatient cases (1000s)	39.1 [30.2 - 51.4]	41.7 [31.7 - 54.3]	47.4 [36.5 - 63.1]
GP referrals (1000s)	79.2 [56.6 - 104.5]	79.9 [56.5 - 106.5]	82.7 [56.8 - 111.7]
Type 1 A&E attendances (1000s)	8.0 [6.5 - 10.6]	8.4 [6.7 - 11.1]	9.3 [7.0 - 12.3]
Type 1 A&E admissions (1000s)	2.4 [1.7 - 3.0]	2.5 [1.9 - 3.2]	2.8 [2.2 - 3.6]
Available general beds	688.0 [467.2 - 918.8]	681.8 [475.4 - 912.6]	663.8 [463.6 - 905.1]
Overnight bed occupancy (%)	90.1 [86.3 - 93.3]	91.0 [86.7 - 93.9]	91.4 [88.0 - 93.9]
Number of consultants	215.1 [154.4 - 344.6]	230.4 [167.9 - 365.0]	246.9 [179.2 - 401.7]
Number of nurses	1257.9 [860.9 - 1800.1]	1246.4 [912.0 - 1781.6]	1323.2 [922.5 - 1906.2]
Number of managers	6576.0 [4733.3 - 9288.3]	6965.7 [4993.4 - 10182.9]	7934.3 [5706.2 - 12078.3]
Number of doctors	524.8 [394.6 - 837.6]	555.9 [426.3 - 842.1]	605.6 [454.1 - 975.5]
Patients from deprivation quintiles Q1/Q2 (%)	45.3 [29.4 - 56.9]	42.3 [28.4 - 56.3]	42.6 [27.6 - 56.1]
Type 1 A&E breach (%)	10.2 [6.3 - 13.7]	16.1 [10.4 - 20.6]	21.2 [14.8 - 28.1]
SHMI % deviation	0.3 [-4.5 - 7.1]	1.0 [-4.2 - 6.6]	1.6 [-4.1 - 8.0]
Safety incidents (/1000 bed days)	78.0 [65.1 - 86.8]	82.2 [73.0 - 93.5]	48.5 [41.1 - 55.2]
Global Digital Exemplar	*NA*	15.0 (11.1%)	*NA*
Major impact from *Wannacry*	*NA*	25 (18.5%)	*NA*

Supplementary Table 1 – Population characteristics of Acute Trusts across each analysed year, showing median [interquartile range] for continuous variables, and number (percentage) for categorical variables. SCR = Shared Care Record; CDMI = Clinical Digital Maturity Index; A&E = Accident and Emergency; SHMI = Summary Hospital Mortality Index. Co-variates described in Table B.

VARIABLES	EXPLANATION AND DATA SOURCE
**Covariates**	
Foundation Trust status	Semi-autonomous management authority compared to non-Foundation Trusts. This variable may be correlated with governance maturity and relative financial independence in procurement. Binary measure in analyses. [Department of Health 2014] [Data files]
Academic Trust status	These are hospitals that are affiliated with a university, and provide specialist tertiary services, thus experiencing a unique case load (including specialist emergency patients) compared to other Trust structures. Binary measure in analyses. [Data files]
Global Digital Exemplar Status	Small number of Trusts that were supported with specific additional funding and partnership opportunities for digital transformation, selected following national digital maturity assessment. Plausibly correlated with accelerated technology implementation. Binary measure in analyses. [NHS England 2017]
Impact from *Wannacry*	The NHS was severely affected by this global ransomware attack, which locked approximately 80 Trusts and 600 primary care practices out of devices. These included 25 Acute Trusts classified in subsequent National Audit Office review as experiencing major disruption. Binary measure in analyses. [National Audit Office 2017]
Clinical Digital Maturity Index	The CDMI is the national digital maturity measure for secondary care Trusts, measured across surveys in 2015/2016, and 2017. We use this to adjust for digital impact on outcomes. Raw aggregate index score used in analyses. [Martin et al 2019] [Data files]
Elective inpatient cases	Annual caseload of planned, elective admissions, from NHS monthly activity administrative data. Scaled in 1000s in analyses. [Metadata] [Data files]
Emergency inpatient cases	Annual caseload of unplanned emergency admissions. Scaled in 1000s in analyses. [Metadata] [Data files]
General Practice referrals	Annual caseload of GP referrals to all general and acute specialties (excluding outpatient psychiatry and psychotherapy referrals). Scaled in 1000s in analyses. [Metadata] [Data files]
Accident & Emergency Type 1 attendances	Annual emergency attendances to A&E (excluding walk-in centres and minor injuries activity). Scaled in 1000s in analyses. [Metadata] [Data files]
Accident & Emergency Type 1 admissions	Annual emergency admissions from A&E (excluding walk-in centres and minor injuries activity). Scaled in 1000s in analyses. Excluded from multivariable models due to high collinearity with attendance data. [Metadata] [Data files]

Available general beds	Mean operational inpatient beds over analysis period from NHS operational data. Excluded from multivariable models due to high collinearity with activity and attendance data. [Metadata] [Data files]
Overnight bed occupancy %	Percentage of operational beds occupied overnight during analysis period. [Metadata] [Data files]
Staff numbers	Number of employed consultant doctors, all doctors, nurses, and managers. From NHS workforce statistics. Managerial staffing used to reflect administrative and operational capabilities. For clinical staff, only nursing numbers included in models due to high collinearity. [Data files]
Deprivation index of attending population	Indices of Multiple Deprivation are the official statistical representation of relative poverty in small geographic areas. Populations are represented in quintiles, with the first quintile representing the most deprived. [UK national statistics, 2019]. For each analysed period, we represented patient deprivation as percentage of population admitted to each Trust from the two most deprived quintiles. Data derived from NHS Digital Hospital Episode Statistics [Data files]
**Outcomes**	
Accident & Emergency Type 1 breach	Percentage of patients who breach the four-hour national A&E wait time target. [Metadata] [Data files]
Summary Hospital Mortality Index	This reports on Trust-level mortality using a standardized methodology. It is derived from patient-level statistics, and is the ratio of number of patients who die following hospitalisation to the number that would be expected to die based on population data adjusted for characteristics of demographic, diagnoses, and co-morbidity [NHS Digital 2021]. Represented in analyses as absolute percent deviation from 1.0. [Data files]
Patient safety incidents	NHS safety reporting is centralized via the National Reporting and Learning System. We include Trust-level data related to reported patient safety incidents. Gross safety incident count adjusted per 1000 bed days. [Data files]
Patient experience survey	Commissioned by the NHS and the Care Quality Commission, and conducted by IPSOS MORI, we include biennial survey outcomes data from questions related to experiences of access to and quality of urgent and emergency care (n = 6 questions) [NHS Surveys]. An aggregate response score was derived per Trust by taking the mean and scaling out of 100. [Data files]

Supplementary Table 2 – Description of covariates, outcome measures, and data sources. Raw data not reproduced due to terms and conditions of use but can be requested from the described sources.

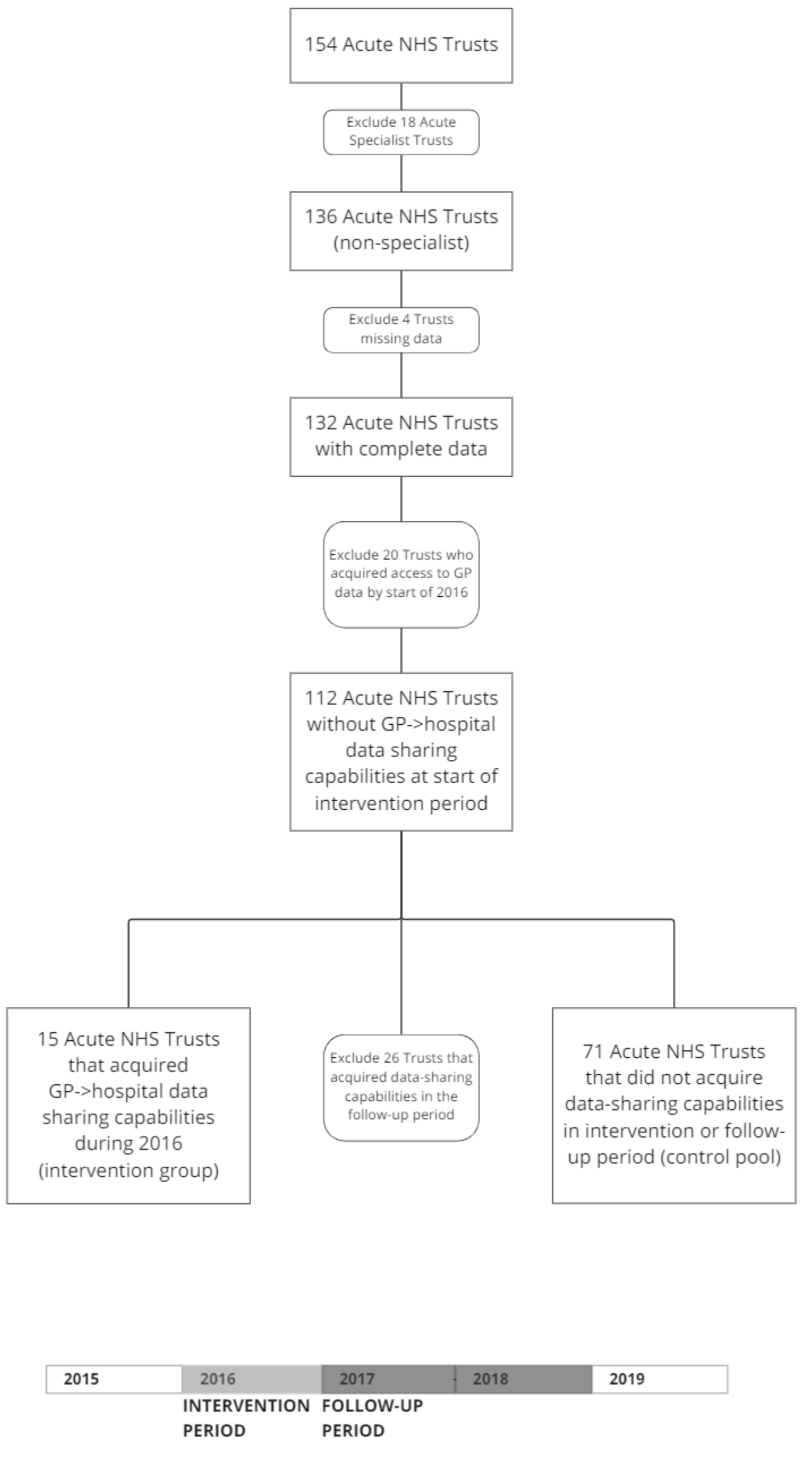

Supplementary Figure 3 – Inclusion flow diagram for Acute NHS Trusts in synthetic control analyses of data-sharing capabilities, showing intervention and follow-up periods. Intervention point is defined as the 31st of December 2016.

	Exposed (n=15)		Placebo (n=71)		
	mean	*std*	*mean*	*std*	*p-val*
2017-01	-0.365	2.035	-0.013	2.827	0.289
2017-02	-0.649	2.472	-0.163	3.168	0.259
2017-03	-0.999	2.653	-0.322	3.481	0.203
2017-04	-1.026	3.028	-0.410	3.842	0.252
2017-05	-1.307	3.218	-0.610	4.151	0.239
2017-06	-1.492	3.443	-0.706	4.395	0.227
2017-07	-1.741	3.249	-0.778	4.481	0.171
2017-08	-1.820	3.233	-0.835	4.660	0.167
2017-09	-2.065	3.385	-0.869	4.788	0.131
2017-10	-2.191	3.374	-0.920	4.828	0.117
2017-11	-2.260	3.321	-0.891	4.850	0.098
2017-12	-2.271	3.371	-0.840	4.846	0.091
2018-01	-2.330	3.474	-0.786	4.813	0.079
2018-02	-2.243	3.716	-0.629	4.818	0.081
2018-03	-2.209	4.051	-0.504	4.891	0.084
2018-04	-2.331	3.843	-0.498	4.948	0.063
2018-05	-2.234	3.829	-0.484	5.117	0.072
2018-06	-2.322	4.047	-0.551	5.195	0.079
2018-07	-2.433	4.270	-0.607	5.379	0.082
2018-08	-2.507	4.472	-0.575	5.463	0.079
2018-09	-2.509	4.669	-0.533	5.662	0.083
2018-10	-2.405	4.874	-0.529	5.926	0.103
2018-11	-2.346	4.849	-0.478	6.098	0.105
2018-12	-2.362	4.685	-0.460	6.230	0.096
2019-01	-2.272	4.641	-0.423	6.504	0.103

Supplementary Table 3 – Results of synthetic control analysis showing average treatment effect and standard deviation (+/-) across exposed Trusts, and control Trusts in 'placebo' analysis, and statistical significance of between group comparison in two-sample, one-tailed t-test.

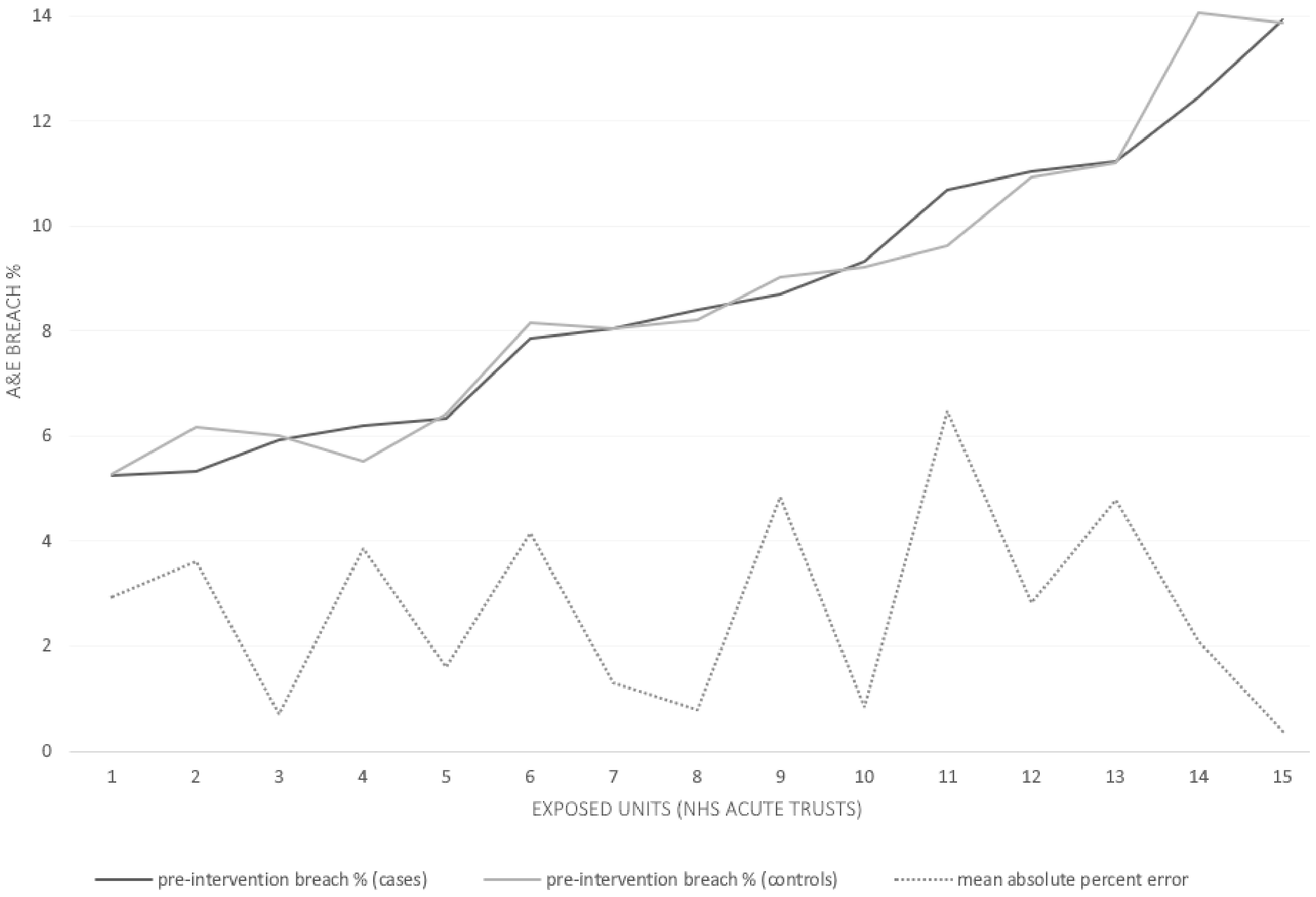

Supplementary Figure 4 – Matching fit for outcome of interest (A&E breach %) across exposed Trusts, showing average pre-intervention breach % for each case (n=15) and corresponding weighted control units, and average forecasting error between cases and weighted controls across entire pre-intervention period.

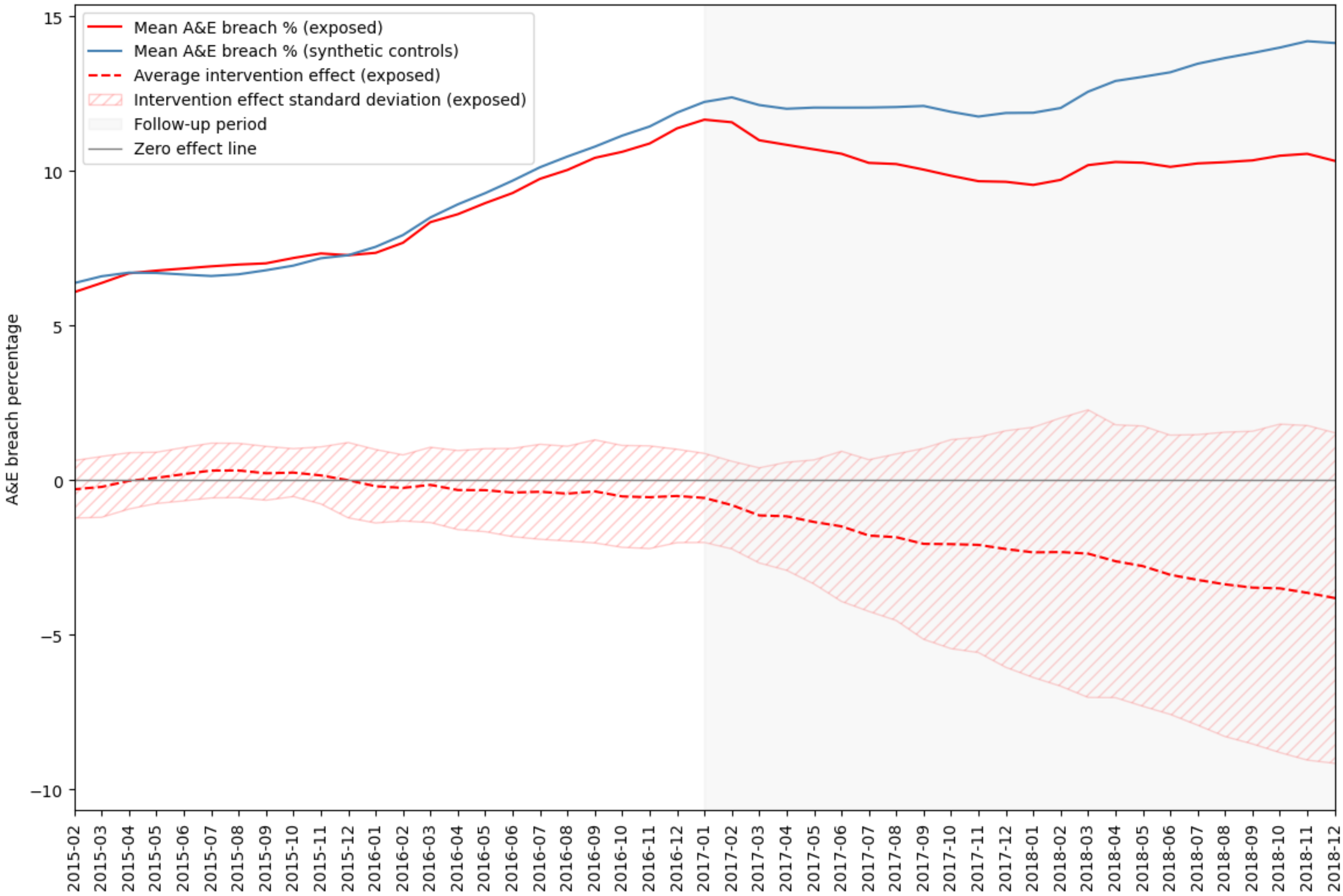

Supplementary Figure 5 – Synthetic control sensitivity analysis with case restriction to Trusts that self-report improved interoperability function. Average A&E breach percentage (n=11, red solid line) vs synthetic controls (blue solid line) constructed from Trusts with no data-sharing capabilities throughout the experimental period (n=71). Average treatment effect in intervention Trusts shown with dotted line (with standard deviation).

	Exposed (n=11)		Placebo (n=71)		
	mean	*std*	*mean*	*std*	*p-val*
2017-01	-0.574	1.441	0.215	2.067	0.066
2017-02	-0.806	1.417	0.230	2.293	0.028
2017-03	-1.139	1.538	0.143	2.550	0.016
2017-04	-1.168	1.748	0.082	2.833	0.030
2017-05	-1.351	2.007	0.006	3.035	0.035
2017-06	-1.493	2.429	-0.067	3.296	0.053
2017-07	-1.791	2.453	-0.138	3.454	0.034
2017-08	-1.845	2.694	-0.143	3.701	0.042
2017-09	-2.059	3.089	-0.140	3.902	0.042
2017-10	-2.069	3.381	-0.195	3.927	0.058
2017-11	-2.092	3.485	-0.214	3.997	0.063
2017-12	-2.227	3.827	-0.242	4.125	0.068
2018-01	-2.335	4.048	-0.299	4.204	0.073
2018-02	-2.325	4.339	-0.282	4.309	0.085
2018-03	-2.375	4.652	-0.140	4.422	0.080
2018-04	-2.621	4.415	-0.083	4.545	0.050
2018-05	-2.780	4.533	-0.121	4.686	0.047
2018-06	-3.059	4.517	-0.149	4.761	0.035
2018-07	-3.226	4.700	-0.176	4.898	0.034
2018-08	-3.370	4.925	-0.101	5.000	0.031
2018-09	-3.475	5.058	0.005	5.214	0.027
2018-10	-3.499	5.313	0.142	5.569	0.027
2018-11	-3.643	5.413	0.279	5.806	0.022
2018-12	-3.812	5.348	0.348	6.107	0.017
2019-01	-3.876	5.614	0.437	6.470	0.018

Supplementary Table 4 – Results of synthetic control sensitivity analysis, including only Trusts with improvement in CDMI self-assessment of interoperability. Shows average treatment effect and standard deviation (+/-) across exposed Trusts, and control Trusts in 'placebo' analysis, and statistical significance of between group comparison in two-sample, one-tailed t-test.

	UNIVARIATE				MULTIVARIABLE				
**Survey A**	*coeff*	*pval*	*ci_lower*	*ci_upper*	*coeff*	*pval*	*ci_lower*	*ci_upper*	VIF
const					79.625	0.000	67.148	92.102	
Data-sharing capability	1.508	0.034	0.114	2.902	1.694	0.015	0.335	3.053	1.21
Global Digital Exemplar	0.698	0.470	-1.208	2.605	0.879	0.409	-1.221	2.978	1.59
CDMI 2017	0.006	0.478	-0.011	0.023	0.006	0.472	-0.011	0.024	1.39
Wannacry impacted	0.412	0.605	-1.161	1.984	0.145	0.842	-1.289	1.579	1.09
Type 1 A&E attendance	-0.320	0.000	-0.460	-0.181	-0.136	0.318	-0.403	0.132	4.13
Bed occupancy (%)	-0.083	0.228	-0.218	0.052	-0.067	0.302	-0.196	0.061	1.17
Emergency activity	-0.053	0.002	-0.086	-0.020	0.010	0.792	-0.066	0.086	6.50
Elective activity	-0.023	0.043	-0.045	-0.001	0.022	0.392	-0.029	0.073	6.91
General & acute referrals	-0.026	0.000	-0.040	-0.013	-0.032	0.028	-0.061	-0.004	5.19
Number of nurses	-0.100	0.014	-0.180	-0.021	0.083	0.440	-0.130	0.296	8.98
Number of managers	-0.007	0.197	-0.017	0.004	-0.002	0.752	-0.016	0.012	2.38
Population deprivation	-0.053	0.001	-0.084	-0.023	-0.056	0.001	-0.088	-0.023	1.36
Foundation Trust status	1.574	0.010	0.377	2.772	1.024	0.084	-0.141	2.189	1.18
Academic Trust status	-1.249	0.115	-2.808	0.309	-1.829	0.088	-3.936	0.279	2.35

Supplementary Table 5 – Results of univariate and multivariable sensitivity analyses of association of factors with improved patient experience of emergency care quality (survey A: 2016/2017), with cases (n=32) restricted to those Trusts self-reporting positive interoperability function in the CDMI survey. Co-variates with substantial multicollinearity (as measured by Variance Inflation Factors (VIF)) are excluded. Co-variates are described in Appendix - Table B. CDMI = Clinical Digital Maturity Index; A&E = Accident and Emergency.

	**UNIVARIATE**				**MULTIVARIABLE**				
**2015**	*coeff*	*pval*	*ci_lower*	*ci_upper*	*coeff*	*pval*	*ci_lower*	*ci_upper*	VIF
const					21.146	0.116	-47.568	5.275	
Data-sharing capability	-0.747	0.782	-6.074	4.581	1.233	0.631	-3.828	6.293	1.10
CDMI 2015/2016	-0.030	0.150	-0.070	0.011	-0.014	0.515	-0.055	0.028	1.26
Type 1 A&E attendance	-0.371	0.060	-0.757	0.016	-0.270	0.434	-0.950	0.411	3.69
Bed occupancy (%)	0.220	0.128	-0.064	0.503	0.295	0.039	0.015	0.575	1.17
Emergency activity	-0.063	0.153	-0.149	0.024	0.034	0.742	-0.170	0.238	6.75
Elective activity	-0.057	0.043	-0.112	-0.002	0.134	0.042	0.005	0.263	6.49
General & acute referrals	-0.050	0.008	-0.087	-0.013	-0.091	0.022	-0.169	-0.014	5.16
Number of nurses	-0.281	0.004	-0.473	-0.089	0.291	0.252	-0.210	0.793	7.84
Number of managers	-0.050	0.000	-0.072	-0.028	-0.057	0.001	-0.088	-0.025	2.16
Population deprivation	-0.014	0.735	-0.093	0.066	0.029	0.479	-0.052	0.111	1.29
Foundation Trust status	-0.581	0.707	-3.634	2.472	-0.250	0.869	-3.248	2.748	1.17
Academic Trust status	-7.034	0.000	-10.687	-3.381	-7.117	0.005	-12.101	-2.134	2.05
**2017**	*coeff*	*pval*	*ci_lower*	*ci_upper*	*coeff*	*pval*	*ci_lower*	*ci_upper*	VIF
const					-19.751	0.226	-51.908	12.407	
Data-sharing capability	-2.782	0.149	-6.571	1.007	-2.304	0.195	-5.803	1.195	1.18
Global Digital Exemplar	-7.781	0.004	-12.962	-2.601	-3.073	0.282	-8.707	2.561	1.56
CDMI 2017	-0.001	0.959	-0.049	0.046	0.018	0.446	-0.029	0.064	4.85
Wannacry impacted	7.766	0.000	3.570	11.961	7.282	0.000	3.429	11.135	1.21
Type 1 A&E attendance	-0.498	0.016	-0.902	-0.094	-0.180	0.642	-0.947	0.587	6.05
Bed occupancy (%)	0.202	0.249	-0.144	0.549	0.224	0.170	-0.098	0.546	6.82
Emergency activity	-0.087	0.055	-0.176	0.002	-0.010	0.919	-0.196	0.177	6.06
Elective activity	-0.078	0.010	-0.137	-0.019	0.104	0.126	-0.029	0.237	8.85
General & acute referrals	-0.064	0.001	-0.103	-0.026	-0.099	0.020	-0.181	-0.016	2.40
Number of nurses	-0.334	0.002	-0.545	-0.124	0.457	0.101	-0.090	1.004	1.35
Number of managers	-0.063	0.000	-0.088	-0.037	-0.074	0.000	-0.110	-0.038	1.15
Population deprivation	0.012	0.791	-0.077	0.101	0.041	0.357	-0.047	0.129	2.29
Foundation Trust status	-0.802	0.642	-4.209	2.606	-0.306	0.844	-3.387	2.775	1.18
Academic Trust status	-8.333	0.000	-12.495	-4.171	-5.015	0.079	-10.629	0.598	1.56
**2019**	*coeff*	*pval*	*ci_lower*	*ci_upper*	*coeff*	*pval*	*ci_lower*	*ci_upper*	VIF
const					-44.41	0.019	-81.400	-7.425	
Data-sharing capability	0.677	0.708	-2.898	4.252	0.600	0.722	-2.739	3.940	1.17
Global Digital Exemplar	-8.502	0.004	-14.211	-2.793	-3.239	0.299	-9.392	2.913	1.46
Type 1 A&E attendance	-0.280	0.143	-0.656	0.096	-0.142	0.706	-0.885	0.602	5.16
Bed occupancy (%)	0.521	0.011	0.122	0.920	0.492	0.014	0.101	0.884	1.22
Emergency activity	-0.049	0.177	-0.121	0.022	0.007	0.930	-0.155	0.169	6.77
Elective activity	-0.073	0.007	-0.125	-0.020	0.070	0.281	-0.058	0.197	7.42
General & acute referrals	-0.055	0.007	-0.094	-0.015	-0.053	0.220	-0.139	0.032	6.09
Number of nurses	-0.265	0.004	-0.447	-0.084	0.218	0.394	-0.287	0.722	9.70
Number of managers	-0.056	0.000	-0.078	-0.035	-0.057	0.001	-0.089	-0.025	2.42
Population deprivation	0.095	0.056	-0.002	0.192	0.112	0.022	0.016	0.207	1.26
Foundation Trust	-0.472	0.794	-4.038	3.095	1.391	0.406	-1.912	4.694	1.15
Academic Trust status	-8.774	0.000	-12.997	-4.550	-6.141	0.046	-12.184	-0.098	2.42

Supplementary Table 6 - Results of univariate and multivariable analyses showing association of data-sharing capabilities and other organisation characteristics with Summary Hospital Mortality Index in 2015, 2017, and 2019, excluding co-variates with high multicollinearity.

	UNIVARIATE				MULTIVARIABLE				
**2015**	*coeff*	*pval*	*ci_lower*	*ci_upper*	*coeff*	*pval*	*ci_lower*	*ci_upper*	VIF
const					134.57	0.000	74.970	194.178	
Data-sharing capability	8.337	0.154	-3.157	19.832	4.375	0.449	-7.041	15.791	1.10
CDMI 2015/2016	-0.011	0.809	-0.100	0.078	-0.048	0.307	-0.142	0.045	1.26
Type 1 A&E attendance	-0.812	0.058	-1.653	0.029	-0.355	0.648	-1.890	1.180	3.68
Bed occupancy (%)	-0.783	0.012	-1.390	-0.175	-0.515	0.109	-1.146	0.116	1.17
Emergency activity	-0.236	0.013	-0.420	-0.052	-0.326	0.163	-0.786	0.133	6.75
Elective activity	-0.071	0.247	-0.192	0.050	-0.010	0.947	-0.301	0.281	6.49
General & acute referrals	-0.082	0.048	-0.163	-0.001	-0.029	0.747	-0.204	0.147	5.16
Number of nurses	-0.112	0.606	-0.543	0.318	0.525	0.359	-0.605	1.656	7.84
Number of managers	-0.008	0.762	-0.059	0.043	-0.019	0.587	-0.090	0.051	2.16
Population deprivation	0.203	0.019	0.034	0.372	0.225	0.017	0.041	0.409	1.29
Foundation Trust status	6.249	0.061	-0.302	12.801	3.035	0.376	-3.729	9.799	1.18
Academic Trust status	5.394	0.202	-2.923	13.711	6.528	0.253	-4.714	17.770	2.05
**2017**	*coeff*	*pval*	*ci_lower*	*ci_upper*	*coeff*	*pval*	*ci_lower*	*ci_upper*	VIF
const					81.860	0.068	-6.062	169.782	
Data-sharing capability	0.754	0.868	-8.229	9.737	-3.850	0.427	-13.417	5.716	1.18
Global Digital Exemplar	2.495	0.696	-10.093	15.083	-0.891	0.909	-16.295	14.513	1.56
CDMI 2017	0.018	0.743	-0.093	0.130	-0.007	0.914	-0.134	0.120	1.36
Wannacry impacted	5.532	0.291	-4.791	15.855	6.335	0.236	-4.200	16.871	1.08
Type 1 A&E attendance	-0.565	0.250	-1.532	0.402	-1.117	0.294	-3.214	0.980	4.85
Bed occupancy (%)	-0.138	0.739	-0.956	0.680	0.060	0.893	-0.821	0.940	1.21
Emergency activity	-0.171	0.110	-0.380	0.039	-0.300	0.246	-0.810	0.210	6.05
Elective activity	-0.068	0.348	-0.209	0.074	-0.268	0.148	-0.631	0.096	6.82
General & acute referrals	-0.020	0.676	-0.113	0.074	0.124	0.277	-0.101	0.350	6.06
Number of nurses	0.054	0.835	-0.459	0.567	1.187	0.119	-0.308	2.682	8.85
Number of managers	-0.008	0.818	-0.073	0.058	-0.031	0.531	-0.131	0.068	2.40
Population deprivation	0.231	0.029	0.025	0.438	0.265	0.031	0.025	0.505	1.35
Foundation Trust status	2.166	0.594	-5.847	10.179	0.659	0.877	-7.764	9.083	1.15
Academic Trust status	4.178	0.426	-6.164	14.521	5.406	0.487	-9.942	20.754	2.29
**2019**	*coeff*	*pval*	*ci_lower*	*ci_upper*	*coeff*	*pval*	*ci_lower*	*ci_upper*	VIF
const					123.57	0.000	71.148	175.997	
Data-sharing capability	-2.208	0.345	-6.813	2.397	-4.849	0.045	-9.583	-0.116	1.17
Global Digital Exemplar	-1.804	0.640	-9.423	5.816	-0.690	0.876	-9.411	8.030	1.46
Type 1 A&E attendance	-0.383	0.120	-0.869	0.102	-0.407	0.446	-1.461	0.647	5.16
Bed occupancy (%)	-0.728	0.006	-1.241	-0.215	-0.729	0.010	-1.284	-0.175	1.22
Emergency activity	-0.085	0.070	-0.177	0.007	-0.118	0.310	-0.348	0.111	6.77
Elective activity	-0.048	0.173	-0.117	0.021	-0.089	0.329	-0.270	0.091	7.42
General & acute referrals	-0.037	0.164	-0.088	0.015	-0.041	0.507	-0.163	0.081	6.09
Number of nurses	-0.049	0.691	-0.291	0.193	1.196	0.001	0.481	1.911	9.70
Number of managers	-0.015	0.333	-0.046	0.016	-0.052	0.025	-0.097	-0.007	2.42
Population deprivation	0.073	0.255	-0.054	0.200	0.021	0.754	-0.114	0.157	1.26
Foundation Trust	1.009	0.665	-3.597	5.616	0.162	0.945	-4.520	4.844	1.15
Academic Trust status	-2.287	0.437	-8.083	3.510	-3.800	0.381	-12.364	4.765	2.42

Supplementary Table 7 - Results of univariate and multivariable analyses showing association of data-sharing capabilities and other organisation characteristics with incidence of patient safety events in 2015, 2017, and 2019, excluding co-variates with high multicollinearity.

**Freedom of Information Questions**
(1) Aside from NHS spine summary care records, are your EHR users able to directly access patient records held by local primary care provider EHRs (e.g. by using an EHR web portal, or healthcare information exchange)?
(1a) If yes, please specify the name and vendor of the current system that enables this
(1b) If yes, when was the current interoperability solution procured?
(2) Aside from NHS spine summary care records (SCR), are your EHR users able to directly access patient records held on a separate hospital Trust's EHR?
(2a) If yes, please specify which Trust's EHR you are functionally interoperable with
(3) In what year was the *first* implementation of an interoperable care record in your Trust? This is defined as a way to electronically access patient data from outside your Trust (e.g. from local GP surgeries, or other local healthcare providers). This does not include access to NHS spine summary care records.
(3a) Please specify the name and vendor (software supplier) for this *first* interoperable Care Record implementation.

Supplementary Table 8 – Freedom of Information questions sent to 152 NHS Trusts in February 2022

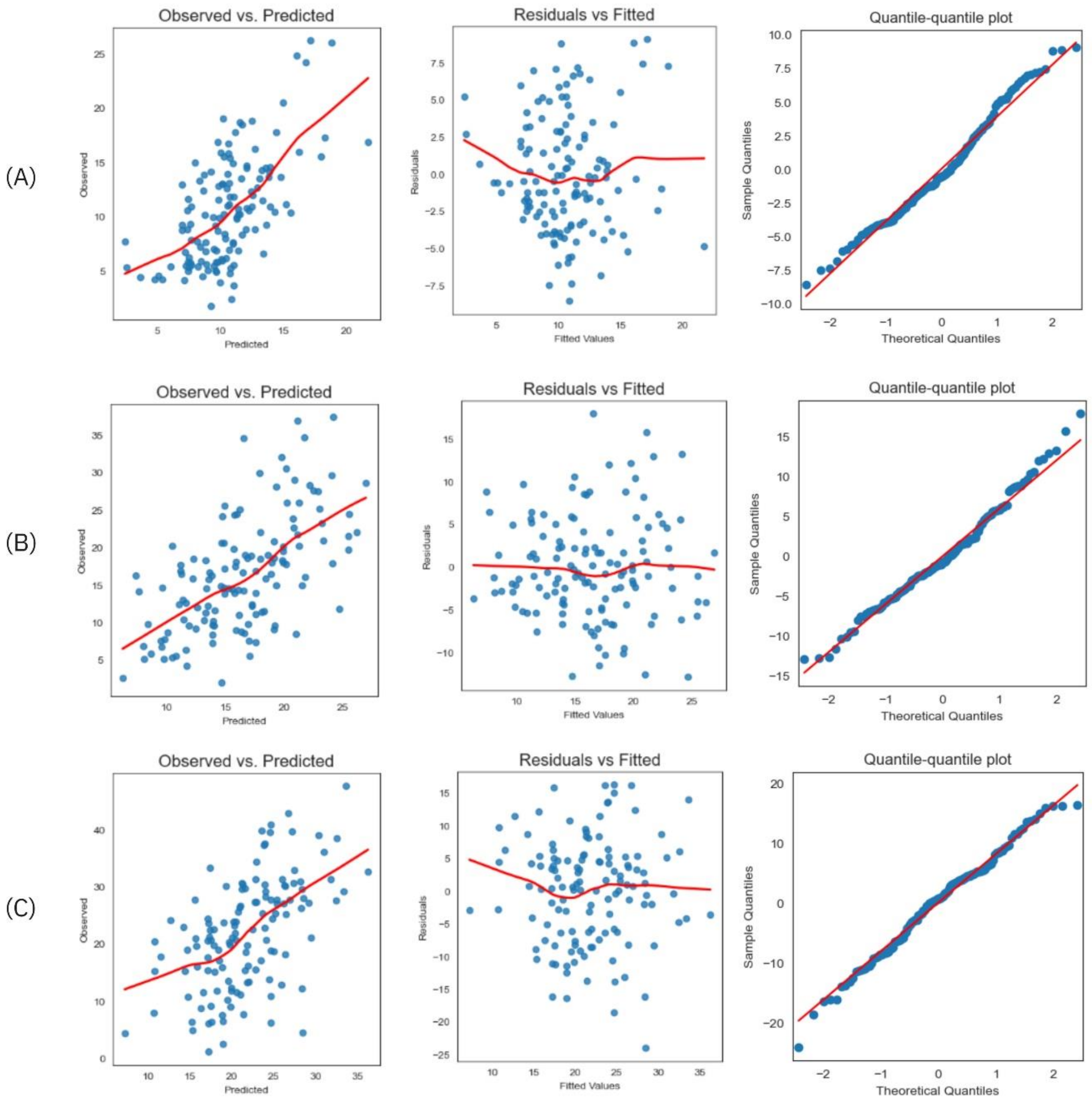

Supplementary Figure 6 – Assessment of A&E breach % multivariable linear regression models for (A) 2015; (B) 2017; (C) 2019

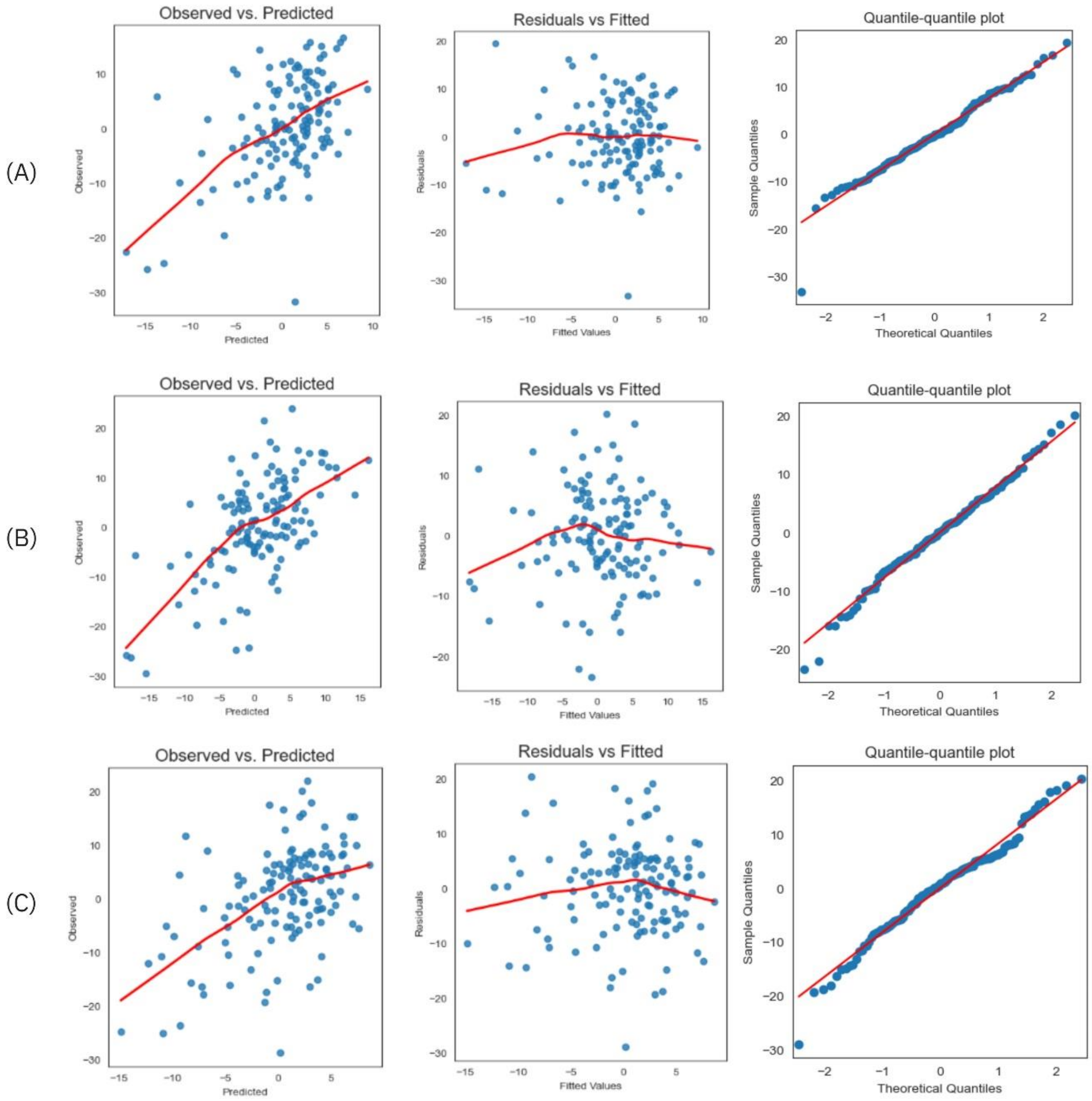

Supplementary Figure 7– Assessment of Summary Hospital Mortality Index multivariable linear regression models for (A) 2015; (B) 2017; (C) 2019

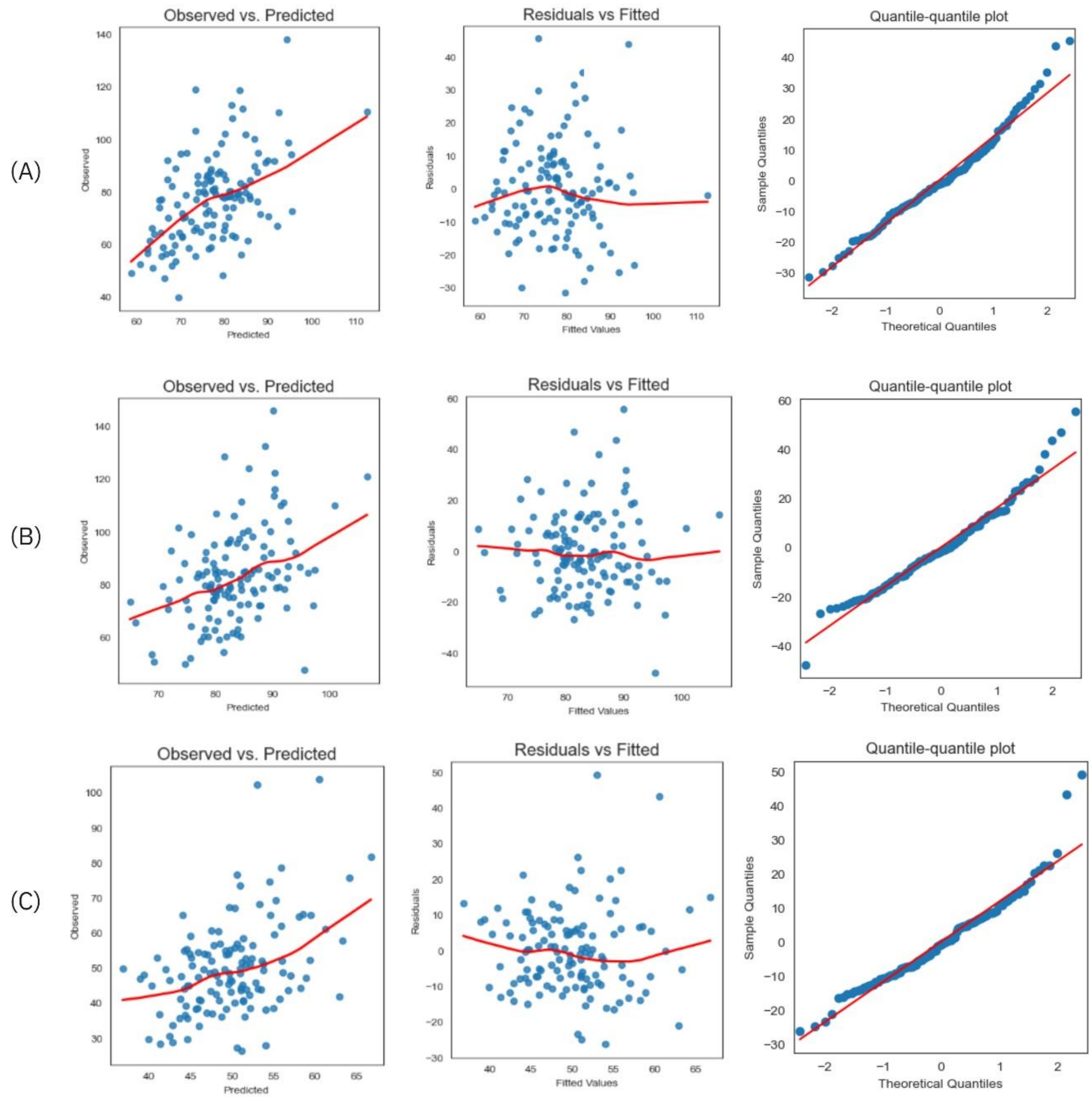

Supplementary Figure 8 – Assessment of patient safety incident multivariable linear regression models for (A) 2015; (B) 2017; (C) 2019

(A)

(B)

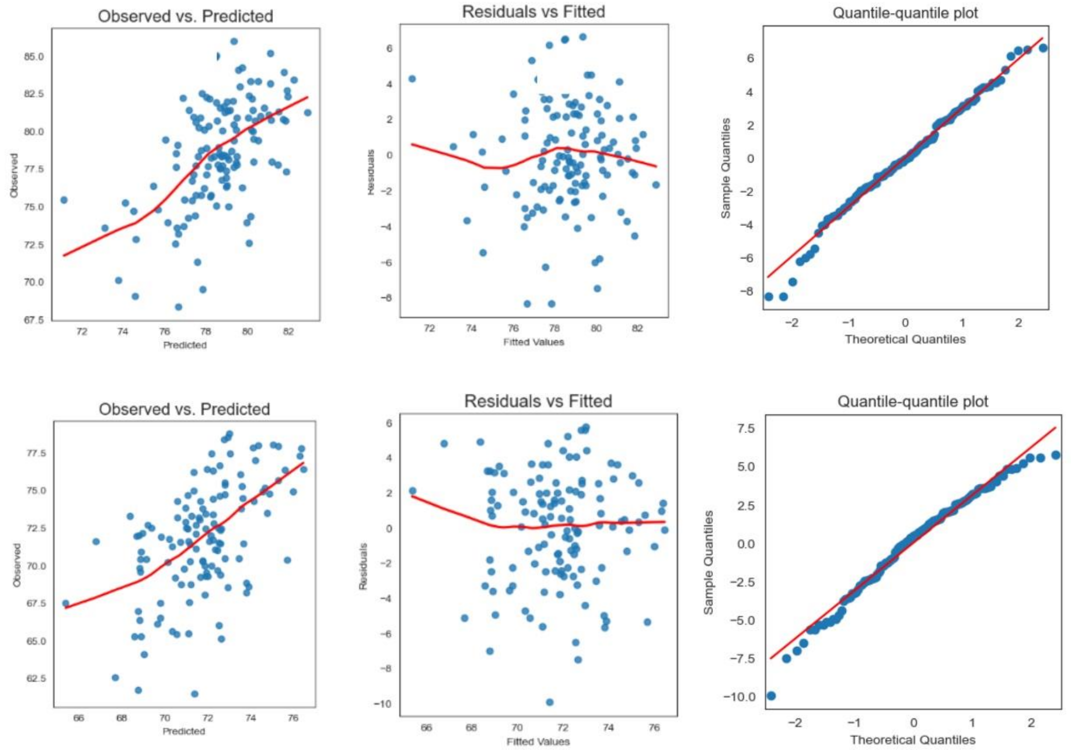

Supplementary Figure 9 – Assessment of patient experience multivariable linear regression models for (A) *Survey* A: 2016/2017; (B) *Survey* B: 2018/2019;

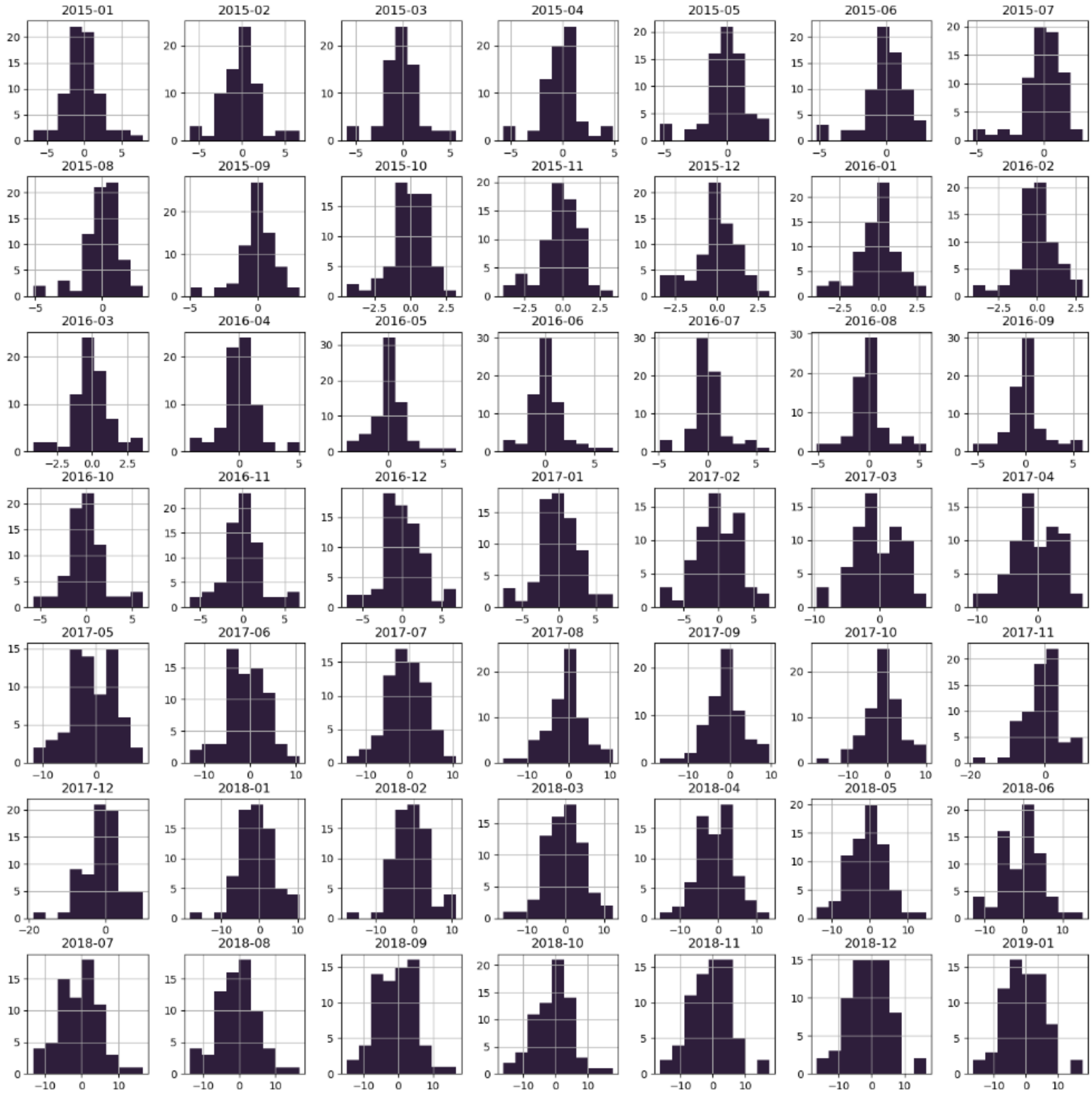

Supplementary Figure 10 – Histogram distribution of average treatment effect at each time point across control units (n=71) in synthetic control experiment.

**Python code packages**	
Data cleaning	*pandas* 1.4.4 [*link*]<br>*numpy* 1.21.5 [*link*]
Multivariable regression analysis	*statsmodels* 0.13.2 [*link*]
Synthetic control analysis	*SyntheticControlMethods* 1.1.17 [*link*]
Visualisation	*seaborn* 0.11.2 [*link*]<br>*Matplotlib* 3.5.2 [*link*]

Supplementary Table 9 – Python packages used in analysis.
